# An Integrative Computational Pipeline for CK2 Inhibitor Discovery in Triple-Negative Breast Cancer Using Virtual Screening, Molecular Dynamics, Machine Learning, and Density Functional Theory

**DOI:** 10.3390/ph19050694

**Published:** 2026-04-28

**Authors:** Abbas Khan, Fahad M. Alshabrmi, Anwar Mohammad, Mohanad Shkoor, Raed M. Al-Zoubi, Long Chiau Ming, Abdelali Agouni

**Affiliations:** 1 Department of Pharmaceutical Sciences, College of Pharmacy, QU Health, Qatar University, Doha P.O. Box 2713, Qatar; abbas.khan@qu.edu.qa; 2 Department of Medical Laboratories, College of Applied Medical Sciences, Qassim University, Buraydah 51452, Saudi Arabia; fshbrmy@qu.edu.sa; 3 Precision Health Analysis Unit, Translational Research, Dasman Diabetes Institute, Dasman 15462, Kuwait; anwar.mohammad@dasmaninstitute.org; 4 Department of Chemistry and Earth Sciences, College of Arts and Science, Qatar University, Doha P.O. Box 2713, Qatar; mshkoor@qu.edu.qa; 5 Surgical Research Section, Department of Surgery, Hamad Medical Corporation, Doha P.O. Box 3050, Qatar; 6 Department of Biomedical Sciences, College of Health Sciences, QU Health, Qatar University, Doha P.O. Box 2713, Qatar; 7 Department of Chemistry, Jordan University of Science and Technology, Irbid 22110, Jordan; 8 School of Pharmacy, Sunway University, Sunway City 47500, Malaysia; chiaumingl@sunway.edu.my

**Keywords:** casein kinase II, machine learning, QSAR, HOMO–LOMO, molecular simulation

## Abstract

**Background:** Triple-negative breast cancer (TNBC) remains among the most aggressive and therapeutically unresponsive subtypes due to the absence of ER, PR, and HER2 targets. Casein Kinase II (CK2), a pleiotropic serine/threonine kinase overexpressed in TNBC, represents a compelling target for rational drug design. **Methods:** Here, we present an AI-integrated benchmarking framework combining virtual drug discovery, molecular dynamics simulations, machine learning-driven QSAR modeling, and quantum-mechanical electronic structure analysis to identify potent CK2 inhibitors from natural product chemical space. **Results:** A validated XP docking protocol (ROC–AUC = 0.748) screened ~480,000 compounds, yielding seven hits, with superior binding to the reference inhibitor CX-4945. Among these, Anastatin B, 3,4,8,9,10-pentahydroxy-dibenzo-[b,d]pyran-6-one, Rhein, and aloe emodin acetate exhibited highly favorable docking scores (−11.6 to −13.1 kcal mol^−1^) and stable 200 ns binding dynamics, reflected by RMSD ≤ 2 Å and compact Rg trajectories. MM-PBSA/MM-GBSA analyses confirmed robust thermodynamic stability, while DFT-derived HOMO–LUMO gaps (3.8–4.3 eV) suggested optimal electronic reactivity for kinase inhibition. Machine learning QSAR models demonstrated strong predictive performance, with the best stacking models achieving test R^2^ ≈ 0.69 and consistent cross-validation performance (CV R^2^ ≈ 0.66–0.69), supporting reliable prediction of pIC_50_ values and prioritization of top-ranked scaffolds. **Conclusions:** Collectively, this integrative framework bridges AI-based learning and biophysical validation, establishing a reproducible paradigm for de novo CK2 inhibitor discovery in TNBC.

## 1. Introduction

Breast cancer is the second most common cancer worldwide, according to the World Health Organization (WHO) [1]. Breast cancer represents the most prevalent malignancy among women and the leading cause of death from cancer in females worldwide [2,3]. There were 2,261,419 new cases of breast cancer worldwide in 2020 (11.7% of all cancers) according to recent data from the Global Cancer Observatory of WHO. Breast cancer caused 6.9% of cancer deaths worldwide in 2020. The incidence and mortality of breast cancer are the highest among other cancers and have been on the rise in recent years, with a higher prevalence in older women. In Qatar, according to recent data from the Global Cancer Observatory in 2020, breast cancer was by far the most common cancer among both sexes, with 14.7% of new cancer cases. In women, breast cancer is also the most common, representing 37.7% of all cancers. It is ranked as the third cause of cancer mortality in Qatar, with 57 (8.1%) deaths after lung (10.9%) and leukemia (8.5%) when both sexes are considered.

Among breast cancer subtypes, triple-negative breast cancer (TNBC) is one of the most aggressive. It accounts for 10–20% of all breast cancer cases [4,5]. TNBCs are defined as breast cancers that do not express human epidermal growth factor receptor 2 (HER2), estrogen receptor (ER), and progesterone receptor (PR) [6]. These cancers are associated with advanced and higher tumor grades, distinct metastasis, and frequent *BRCA1* mutations [4]. The lack of hormone receptors and HER2 expression represents a clinical challenge, as this subtype of breast cancer does not respond to any of the currently available treatments. Therefore, TNBC patients have a tremendously poor prognosis, do not respond satisfactorily to current therapies, and have a remarkably higher relapse rate and disease recurrence compared with non-TNBCs. Currently, there are no approved US Food and Drug Administration (FDA)-targeted medications to manage and treat patients diagnosed with TNBCs. This situation has urged many researchers to address the heterogeneity of TNBC through the identification of novel molecular targets and potential therapeutic options.

CK2 is a serine/threonine-protein kinase that controls key intracellular signaling hubs and is involved in the pathogenesis of many diseases [7]. Emerging evidence suggests that CK2 may be a key molecular effector in breast cancer. CK2 is overexpressed in breast cancer cells, and its activity is associated with increased cell proliferation, migration, and invasion. In contrast, inhibition of CK2’s activity was shown to reduce breast cancer survival. Hence, CK2 has received considerable interest as a therapeutic target in cancer drug design and discovery [7]. In this context, the design and synthesis of bioactive small molecules to inhibit CK2 has gained substantial interest [8]. CK2 has potential as a therapeutic target for cancer as it plays a crucial role in multiple cellular processes, especially for TNBC. Studies have shown that CK2 is often overexpressed and hyperactivated in TNBC, contributing to its aggressive behavior and resistance to standard treatments. Preclinical studies using CK2 inhibitors have shown promising results, including inhibition of tumor growth, induction of cell death, and sensitization of TNBC cells to chemotherapy and radiation therapy. Several CK2 inhibitors reached preclinical and clinical development as potential therapeutic agents. Silmitasertib (CX-4945) is an orally administered CK2 inhibitor that has shown promising results in preclinical studies [9,10,11]. It has demonstrated anti-tumor activity in various cancer types, including breast, prostate, and lung cancers. CX-4945 has undergone Phase I and Phase II clinical trials. In addition, 4,5,6,7-tetrabromobenzotriazole (TBB, NSC 231634) is another CK2 inhibitor, which has been extensively studied in preclinical models and has exhibited potent anti-cancer effects by inhibiting CK2 activity [12]. Quinalizarin (CX-4945) and Ellagic acid (coumarin derivative) are other CK2 inhibitors that have shown inhibitory effects for CK2 and demonstrated potential anti-cancer properties in preclinical studies [13,14]. More potent and innovative therapeutic molecules are required to effectively inhibit this protein in various cancers and overcome the challenge of drug resistance. For instance, computational methods are highly effective in identifying promising hits for clinical applications across diverse diseases [15]. In this context, kinase-driven pathways such as CK2 represent attractive intervention points, and AI-guided drug discovery approaches offer a rational framework to identify potent and selective inhibitors. Leveraging an integrated suite of state-of-the-art computational methodologies, this study combines extra-precision chemical space exploration, long-timescale physics-based simulations, binding free energy calculations, quantum-mechanical analysis, and machine learning-based structure–activity modeling to uncover novel CK2 inhibitors. These approaches collectively enable the identification of potent, selective, and therapeutically safe candidates with strong inhibitory potential against CK2.

## 2. Results and Discussion

### 2.1. Structural Retrieval and Benchmarking

**Figure 1:**
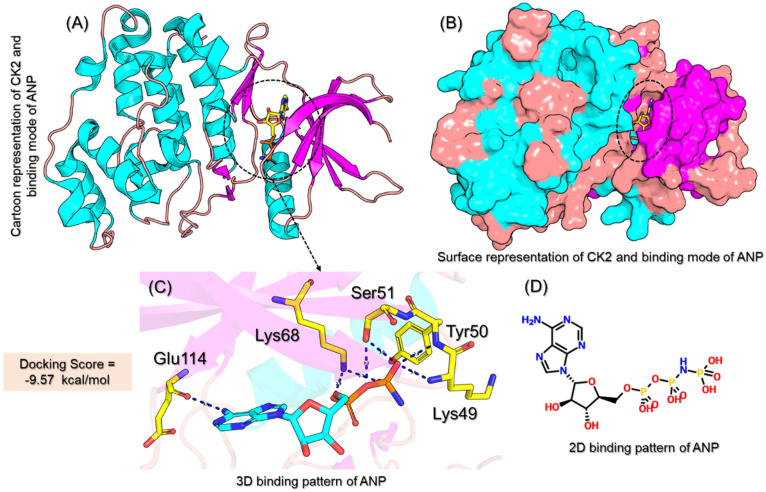
Structural coordinates and binding of CX-4945 with CK2. (**A**) The cartoon representation of CK2 in complex with CX-4945. The bound ligand (CX-4945) is shown in the circle. (**B**) shows the surface representation of the CK2, while (**C**,**D**) show the 3D interaction pattern of CX-4945 with CK2 and a 2D structure of CX-4945.

Casein Kinase 2 is a viable drug target for the discovery of drugs that could inhibit cancer cells in many types of cancers. The CK2 kinase comprises two major regions, i.e., 34–41, necessary for interaction with the beta subunit, and a protein kinase domain spanning from 39–324 residues. The structure of CK2, as given in Figure 1A,B, comprises several helices and beta-sheets that are connected by the surrounding loops. A co-crystal structure of CK2-CX-4945 has been resolved and reported to interact through direct contact by establishing hydrogen bonds with Lys49, Tyr50, Ser51, Lys68, and Glu114 residues from the catalytic domain. Molecular docking of CX-4945 with CK2 revealed a docking score of −9.57 kcal/mol, and the interaction pattern is given in Figure 1C. The 2D structure of CX-4945 is also given in Figure 1D. Although several molecules are available to inhibit CK2 due to a direct and indirect role of CK2 in drug resistance through efflux or DNA repair mechanisms, this has made it challenging to successfully treat cancer. Therefore, more efforts are needed to design more robust binding small-molecule inhibitors that could overcome the mechanism of drug efflux and DNA repair-based resistance.

**Figure 2:**
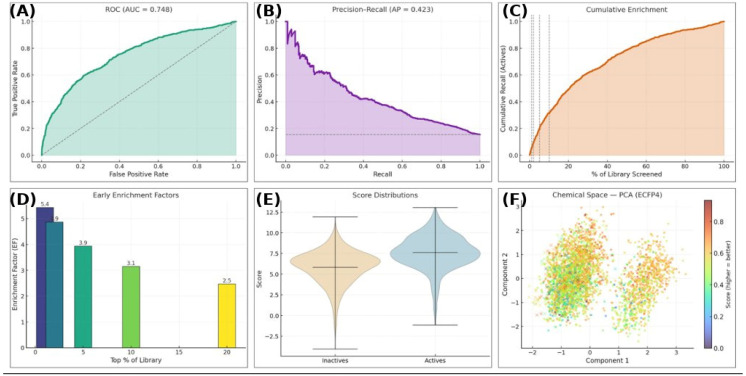
Performance evaluation and chemical space analysis of the CK2 inhibitor classification model. (**A**–**C**) Receiver operating characteristic (ROC, AUC = 0.748) and precision–recall (AP = 0.423) curves demonstrate moderate predictive performance, with cumulative enrichment confirming prioritized retrieval of active compounds early in the screening process. (**D**–**F**) Early enrichment factors (EF_1–20_%) indicate efficient hit recovery within the top-ranked subset of the library. Violin plots show clear separation in score distributions between active and inactive molecules, while PCA of ECFP4 fingerprints reveals broad chemical diversity across the screened space, with higher scores clustering in distinct regions.

The discriminatory performance of the XP docking protocol was evaluated using retrospective enrichment analysis against a benchmark set of active compounds and decoys, and the results are summarized in Figure 2A–F. As shown in Figure 2A, the receiver operating characteristic curve yielded an ROC–AUC of 0.748, indicating that the docking protocol achieved clear separation between active and inactive compounds and performed substantially better than random ranking. Because ROC–AUC can overestimate performance in imbalanced datasets, we further assessed early recognition using the precision–recall framework. In Figure 2B, the precision–recall curve produced an average precision (AP) of 0.423, confirming meaningful recovery of active molecules despite the large excess of decoys. The practical value of the docking protocol for virtual screening is further demonstrated by the cumulative enrichment curve in Figure 2C, which rises steeply at the beginning of the ranked list, indicating preferential concentration of active compounds among the top-scoring molecules. Consistent with this trend, the early enrichment factor analysis in Figure 2D showed strong enrichment at low screening fractions, with EF@1% = 5.4, EF@2% = 4.9, EF@5% = 3.9, EF@10% = 3.1, and EF@20% = 2.5. These values indicate that the docking workflow enriched known actives several-fold above random expectation, particularly within the top-ranked subset that would be prioritized for follow-up analysis. Score distribution analysis further supported the discriminatory ability of the protocol. As shown in Figure 2E, active compounds displayed a clear shift toward more favorable docking scores compared with inactive compounds, although some overlap remained, which is expected for docking-based ranking methods. This result indicates that the scoring function captures relevant binding-related features, while also reflecting the inherent limitations of docking in completely separating actives from decoys. Finally, the PCA projection of ECFP4 fingerprints in Figure 2F showed that highly ranked compounds were distributed across multiple regions of chemical space rather than collapsing into a single dense cluster. This suggests that the docking protocol did not merely prioritize one redundant chemotype but instead retained chemical diversity among top-ranked hits, which is advantageous for downstream hit selection and lead optimization. Taken together, Figure 2 demonstrates that the XP docking workflow provides moderate-to-strong discrimination, meaningful early enrichment, and chemically diverse top-ranked candidates, thereby supporting its suitability as a structure-based prioritization tool in the present kinase inhibitor discovery pipeline.

### 2.2. Extra Precision Screening of Small Molecule Libraries

**Figure 3:**
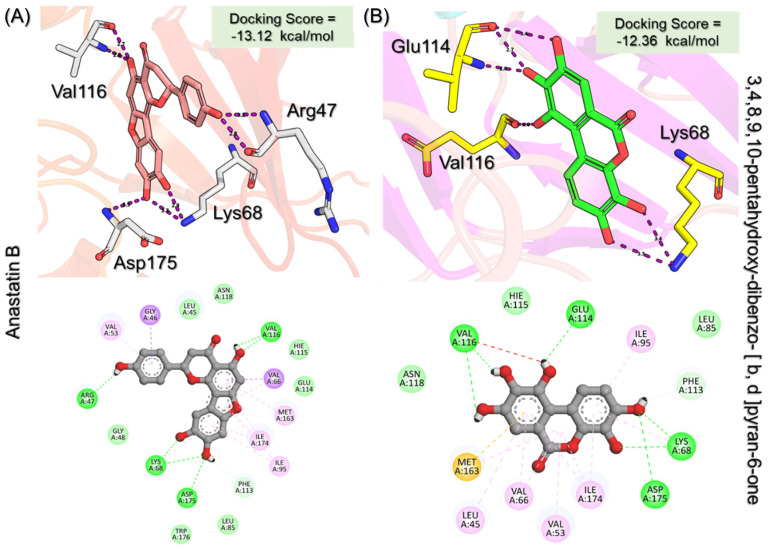
Interaction pattern for the top hits Anastatin B and 3,4,8,9,10-pentahydroxy-dibenzo-[b,d]pyran-6-one. (**A**) shows the 3D and 2D interaction patterns for *Anastatin B,* while (**B**) shows the 3D and 2D interaction patterns for *3,4,8,9,10-pentahydroxy-dibenzo-[b,d]pyran-6-one*. The figures also show the docking score in kcal/mol. Residue interaction maps depict hydrogen bonds as green dashed lines (green nodes) (Pink in 3D interactions), van der Waals contacts as light green nodes, hydrophobic (π–alkyl/alkyl) interactions as pink or purple nodes, π–π stacking or aromatic interactions as orange or yellow nodes, and solvent-accessible regions as blue halos or outlines.

We screened our natural products libraries from South African, North African, Northwest African, and Coconut natural products databases to search for more potent compounds. Each library was subjected to quick preparation by using the QikProp option in the Schrodinger Maestro virtual screening tool. Then, Lipinski’s rule of five filter was applied to filter out the druggable molecules. The three-step extra-precision virtual screening of ~480,000 compounds yielded 151,534 compounds that obey the R5 rule and could be used for further processing. In the high-throughput virtual screening step (HTVS), 2663 compounds were reported to exhibit a pharmacological activity against CK2. These 2663 compounds were then subjected to a standard precision (SP) docking approach, where the top 10% (264) compounds were observed to have better binding potential than the others. In the final stage, extra-precision docking (XP), 29 compounds were obtained as the final best hits. After the removal of duplicates, seven compounds were obtained to have a docking score better than the control (−9.57 kcal/mol). Among these top six hits, Anastatin B reported the best docking score of −13.12 kcal/mol among all. Anastatin B is widely used as a pharmacological agent and has been reported to inhibit liver cancer, cellular malignancies, and mushroom tyrosinase. Anastatin B reported seven hydrogen bonds involving Arg47, Lys68, Val116, and Asp175 as the key interacting residues. In the case of other types of interactions, Gly46, Val53, Val66, Ile95, Met163, and Ile174 are involved to stabilize the binding. Although Anastatin B reported a higher docking score than the control, it can also be seen that the interactions are also conserved as the reported crystalized structure and thus produces similar pharmacological properties by blocking these essential residues. On the other hand, 3,4,8,9,10-pentahydroxy-dibenzo-[b,d]pyran-6-one, which is anthraquinone and possesses hepatoprotective, nephroprotective, anti-cancer, and anti-inflammatory properties, reported a docking score of −12.36 kcal/mol and ranked as the second-best compound among all. Similarly, this compound reported multiple hydrogen bonding interactions with Lys68, Val116, and Asp175 residues, while the other interactions, such as hydrophobic, pie–pie, pie–alkyl, and pie–cation interactions involve Gly46, Val53, Val66, Ile95, Met163, and Ile174 corroborating with the interacting residues of Anastatin B. Interestingly these top hits also strongly align with the interacting residues of CK2 that are previously reported by Sun et al. and others that blocking these residues produces significant pharmacological results [16,17]. This shows that these two compounds possess robust pharmacological potential and, therefore, could be used as a clinical candidate against CK2 in cancer chemotherapy. The 3D and 2D binding modes for Anastatin B and 3,4,8,9,10-pentahydroxy-dibenzo-[b,d]pyran-6-one are given in Figure 3A,B.

**Figure 4:**
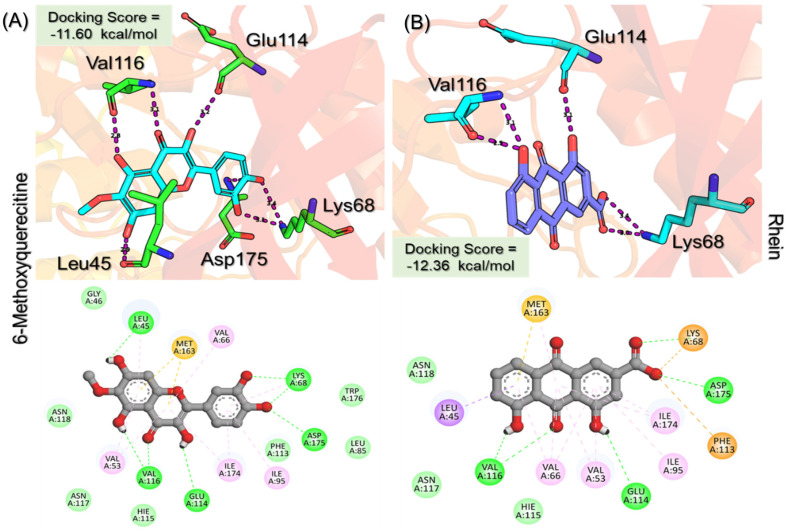
Interaction pattern for the top hits 6-Methoxyquercetin and Rhein. (**A**) shows the 3D and 2D interaction patterns for *6-Methoxyquerecetin* while (**B**) shows the 3D and 2D interaction patterns for *Rhein*. The figures also show the docking score in kcal/mol. Residue interaction maps depict hydrogen bonds as green dashed lines (green nodes) (Pink in 3D interactions), van der Waals contacts as light green nodes, hydrophobic (π–alkyl/alkyl) interactions as pink or purple nodes, π–π stacking or aromatic interactions as orange or yellow nodes, and solvent-accessible regions as blue halos or outlines.

Rhein, which is an anthraquinone that possesses hepatoprotective, nephroprotective, anti-cancer, and anti-inflammatory properties, reported a docking score of **−12.36 kcal/mol** by interacting with the key residues. The interactions involve Lys68, Glu114, Val116, and Asp175 residues and, therefore, block the activity of CK2. Next, we evaluated the binding mode of 6-Methoxyquercetin, which revealed a docking score of **−11.60 kcal/mol** and established several hydrogen and other interactions. A total of seven hydrogen bonds, including Leu45, Lys68, Glu114, Val116, and Asp175, were involved in bonding. On the other hand, Val53, Val66, Lys68, Ile95, Met163, and Ile174 were involved in interactions other than hydrogen bonding. This binding shows that the key residues are blocked by 6-Methoxyquercetin and thus inhibit the activity of CK2 in various cancers. The 3D and 2D binding modes for *6-Methoxyquercetin* and *Rhein* are given in Figure 4A,B.

**Figure 5:**
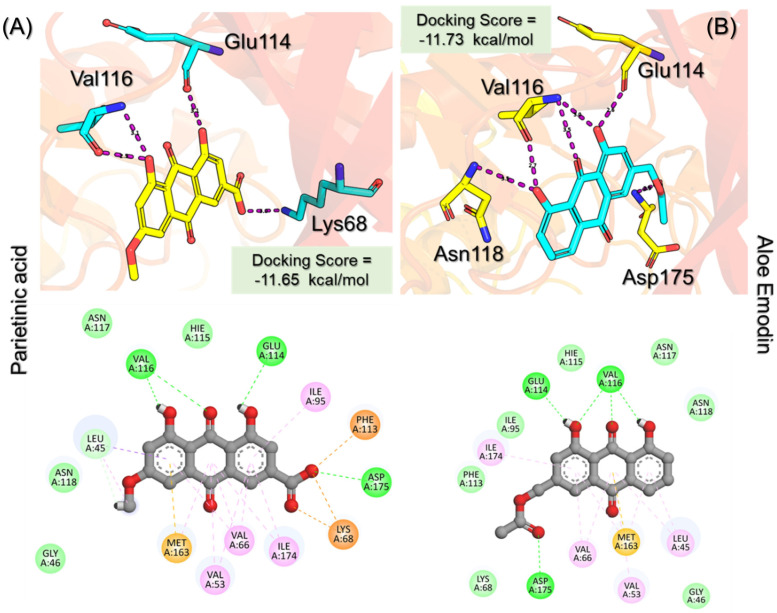
Interaction pattern for the top hits, Parietinic acid and aloe emodin acetate. (**A**) shows the 3D and 2D interaction patterns for Parietinic acid, while (**B**) shows the 3D and 2D interaction patterns for aloe emodin acetate. The figures also show the docking score in kcal/mol. Residue interaction maps depict hydrogen bonds as green dashed lines (green nodes (Pink in 3D interactions)), van der Waals contacts as light green nodes, hydrophobic (π–alkyl/alkyl) interactions as pink or purple nodes, π–π stacking or aromatic interactions as orange or yellow nodes, and solvent-accessible regions as blue halos or outlines.

Parietinic acid demonstrated strong binding affinity toward CK2 with a docking score of −11.65 kcal/mol, forming key interactions within the ATP-binding pocket. Notably, it engaged critical catalytic residues Lys68, Glu114, and Val116, which are known to play essential roles in kinase activity and ligand stabilization. In addition, multiple hydrophobic contacts involving Leu45, Val53, Val66, Ile95, Phe113, Met163, and Ile174 contributed to stabilizing the ligand within the binding cavity, suggesting favorable complementarity activity between the ligand scaffold and the hydrophobic core of the active site. Similarly, aloe emodin acetate exhibited a comparable binding profile with a docking score of −11.73 kcal/mol and formed six hydrogen bonds, primarily involving Glu114, Val116, Asn118, and Asp175. These residues are located within or adjacent to the hinge and catalytic regions of CK2, indicating that the ligand establishes strong polar interactions that are critical for binding specificity. In addition to hydrogen bonding, aloe emodin acetate also formed extensive hydrophobic interactions with residues such as Leu45, Val53, Val66, Lys68, Ile95, Phe113, Met163, and Ile174, reinforcing its stable accommodation within the binding pocket. The interaction patterns of both compounds are illustrated in Figure 5A,B, which depict their 3D binding conformations and 2D interaction maps. These figures confirm that both ligands occupy the canonical active site and engage key residues involved in ATP recognition and catalysis. Complementary structural and interaction details for all top-ranked compounds, along with their docking scores and interacting residues, are summarized in Table 1, providing a comparative overview of binding modes across the ligand set. Importantly, both compounds share structural features that enable consistent engagement with conserved active site residues, suggesting a common binding mechanism. Their docking scores are notably more favorable than those of the reference control ligand, indicating stronger predicted binding affinity. While docking scores alone do not guarantee biological activity, the consistent interaction with key catalytic residues and the convergence of binding modes support their potential as CK2 inhibitors. These compounds were, therefore, selected for further downstream analyses, including molecular dynamics simulations and binding free energy evaluation. Furthermore, the observed interaction profiles are consistent with previously reported CK2 inhibitor binding patterns, where engagement with hinge-region residues such as Glu114 and Val116 is critical for activity. However, the present compounds exhibit enhanced binding scores and comparable or improved interaction networks, suggesting their potential as promising lead candidates for further optimization [18,19,20].

### 2.3. Molecular Simulation-Based Stability Assessment

**Figure 6:**
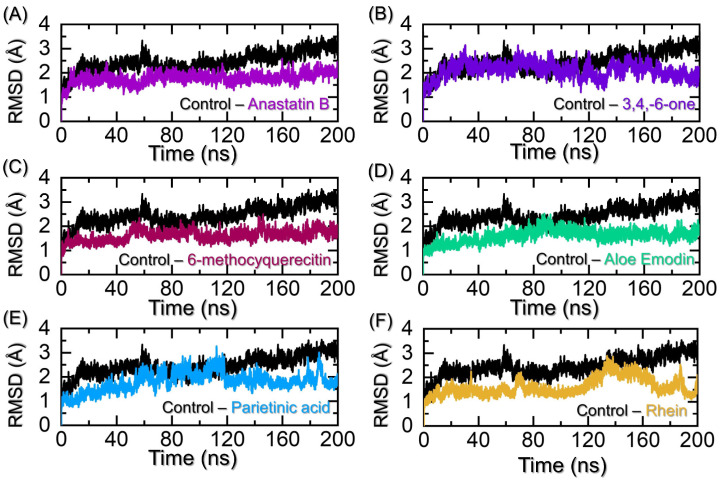
Dynamic stability assessment through RMSD calculation for the top hits against CK2. (**A**) shows the RMSD graphs for the control and Anastatin B–CK2 complexes, (**B**) shows the RMSD graphs for the control and 3,4,8,9,10-pentahydroxy-dibenzo-[b,d]pyran-6-one–CK2 complexes, (**C**) shows the RMSD graphs for the control and 6-methoxyquercetin–CK2 complexes, (**D**) shows the RMSD graphs for the control and aloe emodin–CK2 complexes, (**E**) shows the RMSD graphs for the control and Parietinic acid–CK2 complexes, while (**F**) shows the RMSD graphs for the control and Rhein–CK2 complexes.

To exert the intended pharmacological potential of a drug, dynamic stability is essential. A dynamically stable complex indicates the robust binding of a small molecule and determines the efficacy of the drug. It has been widely used to quantify the dynamic stability as a function of time using the simulation trajectory. Considering the importance of this approach, RMSD, we used the simulation trajectory for each complex, and the stability was assessed. The RMSD for the co-crystallized complex was calculated and used as a reference for the comparative analysis of the top hits. As given in Figure 6A, the control complex reached the equilibrium position at 18 ns and maintained an RMSD of 2.5 Å. Afterward, the production phase started, and the RMSD continued in a uniform pattern. At 60 ns, an abrupt increment was observed where the RMSD level reached up to 3.10 Å. Later, the same pattern was observed with no significant structural perturbation, though the RMSD continued to increase gradually until the end of the simulation. An average RMSD of 2.45 Å was calculated for this complex. In contrast, the Anastatin B–CK2 complex reported lower RMSD values. The RMSD started from 0 and reached a maximum of 2.0 Å at 10 ns, and then a gradual decline in the RMSD was observed. At this point, the RMSD converged with the control complex, thus showing a similar atomic configuration attained by both complexes. A gradual decrease in the RMSD level was observed for the rest of 50 ns, and then after reaching 70 ns. After 70 ns, a smaller increment was observed, and the complex stabilized at 1.80 Å, maintaining the same level until the end of the simulation. An average RMSD for this complex was estimated to be 1.66 Å. It shows that the newly identified molecule, i.e., Anastatin B, is behaving more stable than the control complex and, therefore, produces a stronger pharmacological force than the control, and thus further validates the highest docking score it had in the initial round of screening. Moreover, the RMSD for 3,4,8,9,10-pentahydroxy-dibenzo-[b,d]pyran-6-one reported a similar pattern as the control. The RMSD converged with the control complex with stable dynamic behavior, and with no significant structural perturbations reported. However, the RMSD reported stable dynamics, but still smaller deviations between 100 and 140 ns. The RMSD after 140 ns maintained a lower level than the control. The average RMSD for the 3,4,8,9,10-pentahydroxy-dibenzo-[b,d]pyran-6-one–CK2 complex was calculated to be 1.95 Å. The RMSD graph for the 3,4,8,9,10-pentahydroxy-dibenzo-[b,d]pyran-6-one is given in Figure 6B. Nonetheless, the 6-methoxyquercetin complex also demonstrated lower RMSD values than the control. With no significant structural perturbations in the dynamics condition, the complex equilibrated and stabilized at 1.6 Å at 8 ns. The structure continued to follow the same pattern until 50 ns, and then a small, abrupt increment in the RMSD was observed at 58 ns. For a short period, the RMSD remained higher and then directly decreased and maintained the same level until the end of the simulation. An average RMSD for the 6-methoxyquerecetin–CK2 complex was calculated to be 1.89 Å. The RMSD graph for the 6-methoxyquerecetin–CK2 complex is given in Figure 6C. Similarly, the aloe emodin acetate, when bound to CK2, presented similar dynamic behavior. The RMSD started to incline until 90 ns, and then a uniform straight path was followed. However, the RMSD in the initial states was lower but presented a gradual increment behavior until 90 ns. No significant structural perturbation was experienced, and the complex was observed to be the most stable, with no small deviation. The RMSD graph for the aloe emodin acetate–CK2 complex is given in Figure 6D. On the other hand, the Parietinic acid– and Rhein–CK2 complexes, although demonstrating lower RMSDs, significant structural perturbations were recorded. In the case of Parietinic acid–CK2 complex, the RMSD continued to increase gradually until 120 ns, and then an abrupt decline with minor deviations at different time intervals was observed. The last 10 ns experienced an increased and decreased pattern in the RMSD of the complex. This shows that the binding of these molecules may be released, or internal loops are largely moved, thus causing significant perturbations upon the binding of Parietinic acid. The RMSD graph for the Parietinic acid–CK2 complex is given in Figure 6E. On the other hand, the Rhein–CK2 complex also kept a lower RMSD than the control, but after 140 ns, significant deviations were observed in the RMSD. The RMSD was observed to be lower in the first 140 ns, with a minor deviation at 75 ns, and reached a maximum at 138 ns. The RMSD then decreased back at 170 ns, and then an abrupt increment was experienced. This shows a similar destabilization effect produced by the binding of Rhein to CK2. The RMSD graph for the Rhein–CK2 complex is given in Figure 6F. Overall, these findings demonstrate that our identified novel hits potentially target CK2 more robustly than the control by binding more stable than the control and producing effective pharmacological effects through the blockage of key residues in a dynamic condition. Our results further demonstrate that Anastatin B and aloe emodin acetate are more favorably stable than the other and thus should be prioritized in the experimental testing for clinical applications. For instance, small molecules that bind to CK2 and result in stable dynamic behavior are more pharmacologically active than those that are unstable [21,22].

### 2.4. Structural Compactness Analysis

**Figure 7:**
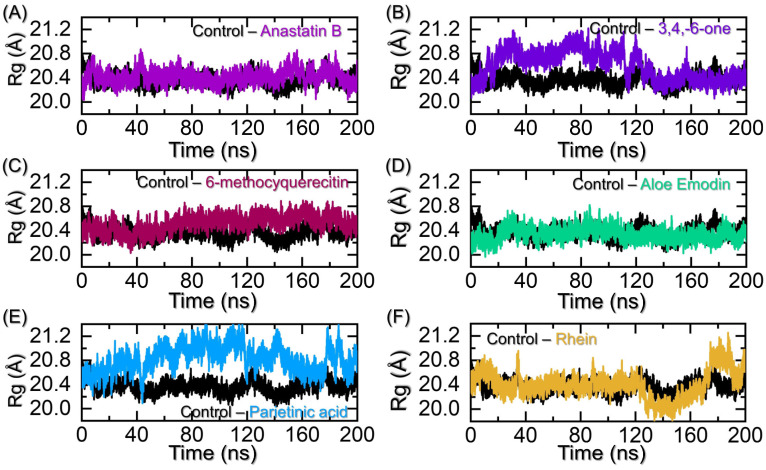
Structural compactness analysis through Rg calculation for the top hits against CK2. (**A**) shows the Rg graphs for the control and Anastatin B–CK2 complexes, (**B**) shows the Rg graphs for the control and 3,4,8,9,10-pentahydroxy-dibenzo-[b,d]pyran-6-one–CK2 complexes, (**C**) shows the Rg graphs for the control and 6-methoxyquercetin–CK2 complexes, (**D**) shows the Rg graphs for the control and aloe emodin–CK2 complexes, (**E**) shows the Rg graphs for the control and Parietinic acid–CK2 complexes while (**F**) shows the Rg graphs for the control and Rhein–CK2 complexes.

Estimation of the size of the protein/compactness reveals significant information regarding the binding and unbinding of the small molecules in a cavity. It is an essential approach to determine the behavior of the protein in the apo and holo states. It has been widely used to extract essential features that are necessary for the pharmacological inhibition of a particular target, specifically the binding and unbinding events. Considering the essential role of Rg in reflecting the potential of small molecules, we also used this approach as a function of time to understand the protein’s compactness. As given in Figure 7A, the control complex, i.e., CK2–Egallic acid, reported an average Rg of 20.60 Å with no significant deviation throughout the simulation. It shows that the complex keeps a stable state with minimal unbinding events and thus demonstrates the robust pharmacological features of this compound. On the other hand, Anastatin B in complex with CK2 reported a similar behavior in terms of structural compactness. No significant deviation was observed, and an average Rg of 20.60 Å was also calculated here. The Rg graphs for the control and Anastatin B strongly corroborate with the RMSD results and thus show uniform dynamic behavior. The Rg results for the control and Anastatin B bound to CK2 are given in Figure 7A. On the other hand, the 3,4,8,9,10-pentahydroxy-dibenzo-[b,d]pyran-6-one–CK2 complex initially reported a higher Rg value, from 10–110 ns, and then the Rg values decreased and converged with the control complex. This loss of compactness is due to the N-terminus and C-terminus corresponding to regions 37–96 and 321–327 amino acids. The N-terminus is particularly important where the binding of a small molecule takes place. Upon the outward movement, the drug is released, which is due to the weaker binding of the drug to hold the N-terminus tighter and thus releases outside. An average Rg for the 3,4,8,9,10-pentahydroxy-dibenzo-[b,d]pyran-6-one–CK2 complex was calculated to be 20.75 Å, which is higher than the control and Anastatin B complexes. The Rg results for the 3,4,8,9,10-pentahydroxy-dibenzo-[b,d]pyran-6-one–CK2 complex are given in Figure 7B. The 6-methoxyquerecitin reported similar behavior as the RMSD and kept a tighter packing of the protein due to the robust binding of 6-methoxyquerecitin. After 120 ns, the Rg increased a little, but that is due to the C-terminus movement, and hence shows minimal unbinding events experienced by this complex. An average Rg for the 6-methoxyquerecitin–CK2 complex was calculated to be 20.65 Å. The Rg results for the 6-methoxyquerecitin–CK2 complex are given in Figure 7C. The Rg results for the aloe emodin acetate–CK2 complex also demonstrated similar behavior as the RMSD. With an increment in the start of the trajectory, the Rg pattern super-aligns with the control, resulting in RMSDs with no significant increase or decrease in Rg values. It is thus showing stable binding of aloe emodin acetate with CK2 and reports an average Rg of 20.57 Å. The Rg graph for the aloe emodin acetate–CK2 complex is given in Figure 7D. Unlike the aloe emodin acetate, the Parietinic acid and Rhein reported fluctuations in the Rg pattern. The Rg of the Parietinic acid–CK2 complex reported significant structural unwinding and causes an increase in protein size. It was also observed that the N-terminus opens up and releases the drug, which consequently causes the lower binding and affinity for Parietinic acid. The Rg graph for the Parietinic acid–CK2 complex is given in Figure 7E. On the other hand, though the Rg for the Rhein–CK2 complex remains lower during the first 1–120 ns, a decreased/increased pattern was observed. An average Rg for the Rhein–CK2 complex was calculated to be 20.68 Å. The Rg graph for the Rhein–CK2 complex is given in Figure 7F. Overall, these findings show that some of these compounds produce stronger pharmacological potentials by showing minimal unbinding events, while some are released from the cavity due to the N-terminus movement, and, therefore, Anastatin B, 6, Methoxy-quercetin, and aloe emodin acetate are the best hits that should be prioritized in clinical testing against CK2 in breast cancer. Our results strongly surpass the previous results, where the maximum unbinding events were reported; however, the results demonstrated that these compounds in complex with CK2 demonstrate stable compact packing of the protein and, therefore, act as stronger pharmacological candidates than the previously reported ones [18].

### 2.5. Root Mean Square Fluctuation Analysis (RMSF)

**Figure 8:**
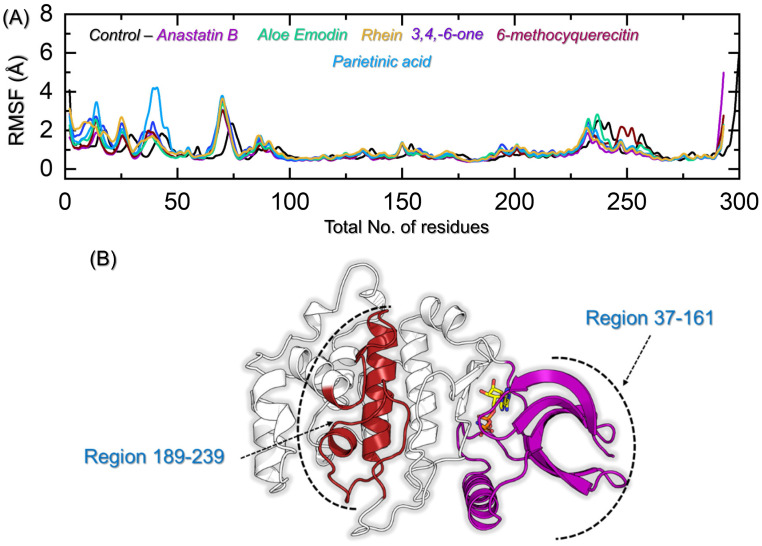
Residue fluctuation indexing in a dynamic environment. (**A**) shows the RMSF for all the complexes, while (**B**) shows the highly dynamic regions in the proteins during the simulation. Residue-wise fluctuations were calculated from the equilibrated MD trajectories to evaluate local flexibility and stability across the protein structure. Ligand-bound systems exhibit reduced fluctuations in key functional regions compared to the control, indicating enhanced structural stabilization upon binding. The highlighted regions correspond to flexible domains that play a critical role in conformational adaptability and ligand interaction dynamics.

Residue fluctuation indexing is an essential parameter that is helpful in molecular recognition, drug discovery, catalysis, and other biological functions. Understanding the flexibility of each residue is essential in the process of drug discovery. Therefore, in the current study, we also used the simulation trajectories to calculate the residues’ flexibility. As shown in Figure 8A, region 1–75, which corresponds to (37–111), demonstrated higher fluctuations. This is a domain that is connected to the other part through a loop, and, therefore, the flexibility increases the movement of this loop and causes higher fluctuation. In the different regions, such as 76–225, these presented more alike with minimal fluctuations. The region 226–260 corresponds to a loop and a helix that covers the active site residues of the catalytic domain and causes opening and closing that help in pushing the drug in and out during simulation. The regions that fluctuate more frequently are highlighted in Figure 8B. In sum, the binding of different drugs, such as the control, Anastatin B, 6, Methocyquerecitine, and aloe emodin acetate, reduces the internal fluctuation of region 37–161 and stabilizes the internal residues’ fluctuation, consequently producing the pharmacological effects.

### 2.6. Hydrogen Bonding Analysis

**Figure 9:**
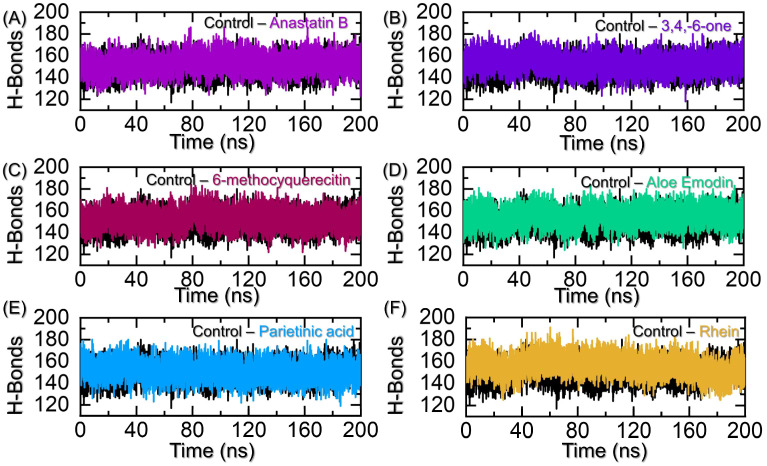
Hydrogen bonding graphs for the top hits against CK2. (**A**) shows the H-bond graphs for the control and Anastatin B–CK2 complexes, (**B**) shows the H-bond graphs for the control and 3,4,8,9,10-pentahydroxy-dibenzo-[b,d]pyran-6-one–CK2 complexes, (**C**) shows the H-bond graphs for the control and 6-methoxyquercetin–CK2 complexes, (**D**) shows the H-bond graphs for the control and aloe emodin–CK2 complexes, (**E**) shows the H-bond graphs for the control and Parietinic acid–CK2 complexes, while (**F**) shows the H-bond graphs for the control and Rhein–CK2 complexes.

Macromolecular complexes, particularly protein coupling with small molecular or another protein partner, are primarily driven by hydrogen bonding and hydrophobic contacts. The environment of protein interfaces is enriched with water molecules that work with the residues to form hydrogen bonds. Thus, it is important to understand the hydrogen bonding landscape in a molecular association. For instance, previously, hydrogen bonding was predicted to estimate the strength of the association between two molecules, which shed light on different mechanisms. Here, we have employed a similar approach to understand the differences in hydrogen bonding between the control and the bound lead molecule complexes. The average number of hydrogen bonds was higher for Anastatin B, aloe emodin acetate, and Rhein than for the control. While for the others, during the simulation, the number of hydrogen bonds is less than that of the control. Hence, this further confirms the robust pharmacological potential of Anastatin B, aloe emodin acetate, and Rhein in contrast to the control drug. In conclusion, our results report Anastatin B, aloe emodin acetate, and Rhein as the promising inhibitors for CK2 activity in breast cancer. The hydrogen bonding results are given in Figure 9A–F.

### 2.7. Binding Free Energy Calculation

To re-evaluate the binding conformation, we used the binding free energy calculation approach using both the MM-PBSA and MM-GBSA methods. These methods are highly applicable, accurate, fast, and reliable in terms of evaluating the activity of a small molecule that is predicted by docking simulation. This approach has been used for years to design novel inhibitors for various targets in different diseases such as cancer, diabetes, COVID-19, monkeypox virus, etc. Considering the higher applicability of these methods, we also utilized them to re-evaluate the binding potential of our top hits from docking. The binding free energy was calculated for different time intervals: 1–10 ns (equilibrium state), 11–30 ns (region of observed de-stability), and 185–200 ns, which represented the most stable phase of all complexes. The MM-PBSA results for the control drug during 1–10 ns revealed a van der Waals (vdW) contribution of −33.58 ± 0.15 kcal/mol, while Anastatin B exhibited a stronger vdW energy of −42.78 ± 0.32 kcal/mol. Aloe emodin acetate, 6-methoxyquercetin, and the newly engineered scaffold showed vdW values of −39.02 ± 0.13 kcal/mol, −38.41 ± 0.15 kcal/mol, and −38.39 ± 0.31 kcal/mol, respectively. The compounds 3,4,8,9,10-pentahydroxy-dibenzo-[b,d]-pyran-6-one, Parietinic acid, and Rhein demonstrated comparatively lower vdW values of −24.99 ± 0.19 kcal/mol, −29.14 ± 0.11 kcal/mol, and −27.27 ± 0.29 kcal/mol, respectively. Similar vdW energies were also observed from the MM-GBSA method. For the electrostatic energy component (EEL) within 1–10 ns, the control reported −21.62 ± 0.31 kcal/mol, whereas the top hits displayed values of −9.12 ± 0.21 kcal/mol for Anastatin B, −5.97 ± 0.21 kcal/mol for aloe emodin acetate, −122.47 ± 0.53 kcal/mol for Parietinic acid, −9.12 ± 0.18 kcal/mol for 3,4,8,9,10-pentahydroxy-dibenzo-[b,d]-pyran-6-one, −13.47 ± 0.20 kcal/mol for 6-methoxyquercetin, and −175.31 ± 1.46 kcal/mol for Rhein. The total binding free energy (TBE) during 1–10 ns confirmed that the control drug had a TBE of −15.52 ± 0.16 kcal/mol, while Anastatin B recorded −26.85 ± 0.20 kcal/mol, aloe emodin acetate −17.28 ± 0.10 kcal/mol, Parietinic acid −16.37 ± 0.15 kcal/mol, 3,4,8,9,10-pentahydroxy-dibenzo-[b,d]-pyran-6-one −16.37 ± 0.15 kcal/mol, 6-methoxyquercetin −23.95 ± 0.18 kcal/mol, and Rhein −14.82 ± 0.37 kcal/mol. The corresponding MM-GBSA TBEs were −16.29 ± 0.16 kcal/mol for the control, −29.23 ± 0.20 kcal/mol for Anastatin B, −33.36 ± 0.17 kcal/mol for aloe emodin acetate, −16.15 ± 0.89 kcal/mol for Parietinic acid, −12.91 ± 0.15 kcal/mol for 3,4,8,9,10-pentahydroxy-dibenzo-[b,d]-pyran-6-one, −23.86 ± 0.17 kcal/mol for 6-methoxyquercetin, and −17.44 ± 0.66 kcal/mol for Rhein. During the 11–30 ns interval, the MM-PBSA results revealed vdW energies of −34.85 ± 0.13 kcal/mol for the control, −43.68 ± 0.14 kcal/mol for Anastatin B, −38.83 ± 0.08 kcal/mol for aloe emodin acetate, −30.65 ± 0.10 kcal/mol for Parietinic acid, −21.21 ± 0.18 kcal/mol for 3,4,8,9,10-pentahydroxy-dibenzo-[b,d]-pyran-6-one, −37.02 ± 0.12 kcal/mol for 6-methoxyquercetin, and −24.19 ± 0.15 kcal/mol for Rhein. The corresponding EEL values were −24.13 ± 0.25 kcal/mol for the control, −8.22 ± 0.10 kcal/mol for Anastatin B, −5.97 ± 0.21 kcal/mol for aloe emodin acetate, −125.01 ± 0.61 kcal/mol for Parietinic acid, −7.09 ± 0.12 kcal/mol for 3,4,8,9,10-pentahydroxy-dibenzo-[b,d]-pyran-6-one, −12.83 ± 0.11 kcal/mol for 6-methoxyquercetin, and −169.14 ± 1.13 kcal/mol for Rhein. The total binding free energy for 11–30 ns was −16.14 ± 0.12 kcal/mol for the control, −27.32 ± 0.10 kcal/mol for Anastatin B, −26.62 ± 0.13 kcal/mol for aloe emodin acetate, −17.58 ± 0.13 kcal/mol for Parietinic acid, −14.17 ± 0.12 kcal/mol for 3,4,8,9,10-pentahydroxy-dibenzo-[b,d]-pyran-6-one, −23.16 ± 0.10 kcal/mol for 6-methoxyquercetin, and −15.01 ± 0.16 kcal/mol for Rhein. Despite minor declines in stability for certain complexes, Anastatin B continued to display an increasing TBE, highlighting its stronger pharmacological potential against CK2. In the final stabilized phase (185–200 ns), the control complex showed a TBE of −16.51 ± 0.08 kcal/mol, while Anastatin B maintained the highest stability with a TBE of −27.84 ± 0.08 kcal/mol. Aloe emodin acetate exhibited a TBE of −24.13 ± 0.08 kcal/mol, Parietinic acid –22.34 ± 0.11 kcal/mol, 3,4,8,9,10-pentahydroxy-dibenzo-[b,d]-pyran-6-one −14.15 ± 0.07 kcal/mol, 6-methoxyquercetin −19.07 ± 0.07 kcal/mol, and Rhein −14.53 ± 0.04 kcal/mol. Using the MM-GBSA method, the corresponding TBEs were −17.25 ± 0.08 kcal/mol for the control, −27.97 ± 0.07 kcal/mol for Anastatin B, −27.47 ± 0.07 kcal/mol for aloe emodin acetate, −22.78 ± 0.91 kcal/mol for Parietinic acid, −11.68 ± 0.63 kcal/mol for 3,4,8,9,10-pentahydroxy-dibenzo-[b,d]-pyran-6-one, −18.42 ± 0.05 kcal/mol for 6-methoxyquercetin, and −13.15 ± 0.15 kcal/mol for Rhein. Overall, the progressive strengthening of Anastatin B’s binding energy across time intervals demonstrates its sustained interaction and enhanced stability within the CK2 binding pocket. Aloe emodin acetate and 6-methoxyquercetin also exhibited strong binding comparable to the control, indicating that these molecules could serve as promising CK2 inhibitors with therapeutic potential, pending further pharmacological validation. Our compounds report better binding free energies than the previously reported compounds, further validating the robust binding in contrast to the previously discovered molecules [21,23,24,25,26]. All the results, including the vdW, EEL, PB, GB, Delta G gas, Delta G solvated, and the total binding free energies, are given in Table 2 and Table 3. All the energies are given in kcal/mol.

### 2.8. Frontier Molecular Orbital (FMO) and Electronic Structure Analysis

The analyzed compounds exhibited distinct HOMO–LUMO profiles that reflect their stability and electronic transitions. Parietinic acid displayed a HOMO at −6.85 eV, a LUMO at −3.05 eV, and a gap of 3.80 eV, suggesting moderate stability and potential reactivity through π–π interactions. Its relatively strong dipole moment (2.36 D total) indicates favorable polarity for biomolecular interactions. Rhein exhibited a slightly wider gap (3.86 eV) with a more negative HOMO (−7.02 eV), suggesting higher oxidative stability but reduced electron-donating ability compared to Parietinic acid. 6-Methoxy quercetin and 3,4,8,9,10-pentahydroxy-dibenzo-[b,d]pyran-6-one both showed narrower band gaps (4.21 and 4.37 eV, respectively), reflecting higher electronic polarizability. Their higher LUMO levels (−1.36 and −1.28 eV) indicate stronger electron-accepting potential, which may enhance radical scavenging and antioxidant activity. The dipole moment of the pentahydroxy derivative (8.39 D) was particularly large, suggesting pronounced polarity and solubility, factors relevant for pharmacological interactions. Anastatin B exhibited a HOMO of −5.45 eV and a LUMO of −1.27 eV, yielding a gap of 4.18 eV. Its electronic properties, combined with a substantial dipole moment (3.62 D), suggest enhanced reactivity and intermolecular binding potential. In contrast, aloe emodin revealed a HOMO at −6.85 eV and a LUMO at −2.86 eV, with a gap of 3.99 eV. Its intermediate dipole moment (2.70 D) and electronic gap suggest a balanced profile between stability and reactivity, consistent with the reported pharmacological versatility of anthraquinones. Overall, the HOMO–LUMO analysis highlights subtle variations in electronic structure among these polyphenolic compounds. Narrower band gaps and higher dipole moments correspond to enhanced electron transfer and interaction potential, supporting their reported antioxidant and bioactive properties. The combination of orbital mapping, energy gap analysis, and dipole evaluation provides valuable insight into the electronic factors underpinning their biological functions. The results are given in (Supplementary Figure S1).

To complement the docking and molecular dynamics results, density functional theory (DFT) calculations were employed to examine the frontier molecular orbitals (FMOs) of the selected scaffolds. Both the highest occupied molecular orbital (HOMO) and lowest unoccupied molecular orbital (LUMO) were analyzed to gain insights into electron distribution, reactivity, and possible interaction sites with the Casein Kinase II active site (Supplementary Figure S2). For the representative scaffold, the HOMO energy was calculated at −7.02 eV, while the LUMO energy was found at −3.16 eV, giving a HOMO–LUMO gap (ΔE) of 3.86 eV. This moderate gap reflects a balance between chemical stability and reactivity, making the scaffold electronically suitable for bioactive interactions. Molecules with significantly smaller gaps often display excessive reactivity, whereas excessively large gaps correspond to chemically inert systems. The HOMO density plots were primarily localized on amide, carbonyl, and nitrogen-rich moieties, highlighting their ability to act as electron donors during binding, facilitating hydrogen bonding with polar residues in the ATP-binding cleft. In contrast, the LUMO densities were concentrated on the aromatic π-system, which suggests a strong propensity for π–π stacking interactions with hydrophobic and aromatic residues. In certain scaffolds, dual orbital delocalization was observed, enabling a combined donor–acceptor behavior that strengthens ligand–protein stabilization. The electronic features identified here complement the binding free energy decomposition from MM/GBSA and molecular simulations, where hydrogen bonding and π-stacking were consistently highlighted as key stabilizing interactions. Moreover, the HOMO–LUMO alignment confirms that polar regions drive specificity, while aromatic orbital delocalization drives affinity, offering a quantum-level validation of the observed QSAR and docking trends. Thus, the frontier orbital analysis not only provides quantum-mechanical evidence of electronic suitability but also benchmarks the scaffolds’ bioactivity potential against other computational approaches. This integrated interpretation reinforces their candidacy as potent CK2 inhibitors.

### 2.9. Machine Learning-Based Activity Prediction

Across all descriptor and fingerprint combinations, our AI-driven QSAR framework demonstrated consistent predictive capacity, with several models achieving performance levels indicative of reliable generalization. In response to reviewer concerns, cross-validation metrics (CV R^2^ ± SD) are now explicitly reported for all models (Table 4), enabling a more rigorous assessment of model robustness. Importantly, the relationship between train R^2^, CV R^2^, and test R^2^ across models does not support the presence of pathological overfitting. For the best-performing feature spaces, particularly fingerprint-based (FP) representations, CV R^2^ values closely align with external test R^2^ values. For example, the stacking ensemble on FP features achieved test R^2^ = 0.690 and CV R^2^ = 0.693 ± 0.0036, while Random Forest and Gradient Boosting models yielded test R^2^ values of 0.668–0.664 with corresponding CV R^2^ values of 0.666 ± 0.041 and 0.664 ± 0.043, respectively. This strong agreement between CV and test performance indicates stable model generalization rather than overfitting to the training data. While train R^2^ values are consistently higher than expected for ensemble learners, the observed train–test gaps (ΔR^2^ ~0.13–0.20 for most top-performing models) remain moderate and do not translate into inflated CV performance. Moreover, the relatively low standard deviations in CV R^2^ (typically ~0.03–0.06) demonstrate low variance across folds, further supporting model stability. Tree-based ensemble methods dominated performance across all feature sets. Random Forest, Gradient Boosting, and Histogram Gradient Boosting consistently achieved test R^2^ values in the range of 0.64–0.68, with closely matching CV R^2^ values, confirming their robustness. Fingerprint-derived features (FP) emerged as the most predictive representation, while combined feature spaces (e.g., 2D + FP, 3D + FP, and full integration) further enhanced performance, highlighting the complementary nature of structural and physicochemical descriptors. 

**Figure 10:**
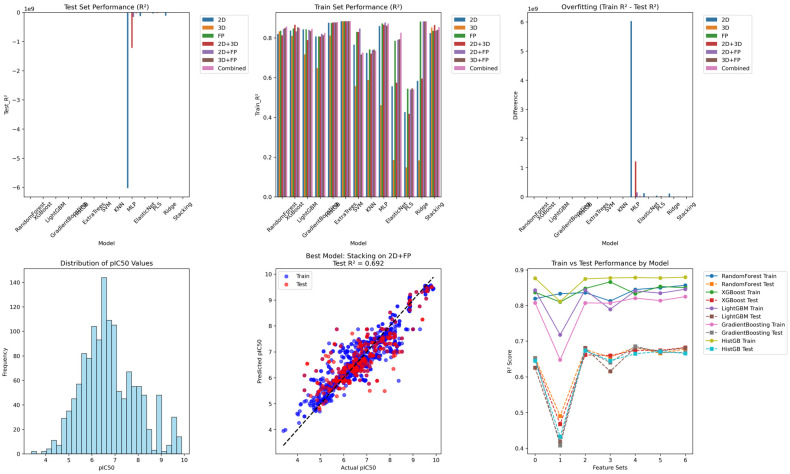
Machine learning model benchmarking for CK2 inhibitor prediction. Comparison of train–test performance (R^2^), generalization gap (ΔR^2^), and pIC_50_ value distribution across 2D, 3D, fingerprint, and combined feature sets. Stacking ensemble models achieved the highest predictive performance, with the combined feature model reaching a **test R^2^ ≈ of 0.69** and consistent cross-validation performance, indicating robust generalization across CK2 inhibitor datasets.

In contrast, 2D and 3D descriptors alone exhibited lower predictive power (test R^2^ of 0.45–0.65), reflecting their limited ability to capture structure–activity relationships fully. Stacking was implemented as a post hoc ensemble strategy to assess whether combining the strongest performing regressors within each feature space could improve predictive stability. The Graph Neural Network (GIN) baseline showed minimal predictive capability (test R^2^ = 0.020), likely due to data size limitations and lack of extensive hyperparameter optimization, further emphasizing the strength of classical ensemble approaches in moderate-sized datasets. Overall, the consistency between CV and external test performance, combined with controlled ΔR^2^ values and low CV variance, demonstrates that the developed QSAR models are robust, generalizable, and not overfitted, supporting their suitability for drug discovery applications. The optimal stacking model (2D + FP) demonstrated robust predictive performance, with a training RMSE of 0.486, a test RMSE of 0.642, and a cross-validation RMSE of 0.652 ± 0.052, consistent with the comprehensive benchmarking results presented in Supplementary Table S1. All the results are given in Table 4, while the machine learning model benchmarking for CK2 inhibitor prediction is shown in Figure 10.

### 2.10. Determining the pIC50 Values via Stacking Ensemble Model

For the CX series, the corresponding IC_50_ values were converted to pIC_50_ using the equation pIC_50_ = −log_10_(IC_50_). The reported pIC_50_ range (5.70–9.52) corresponds to experimentally observed IC_50_ values between 0.3 and 2000 nM, obtained from 23 assays. Similarly, the newly identified hits exhibited comparable predicted IC_50_ ranges. Specifically, the Parietinic acid–CK2 complex showed a pIC_50_ of 7.263, the Rhein–CK2 complex 7.661, 6-methoxyquercetin 7.408, 3,4,8,9,10-pentahydroxy-dibenzo-[b,d]pyran-6-one 8.140, the Anastatin B–CK2 complex 9.376, and the aloe emodin–CK2 complex 7.113. On the other hand, the predicted pIC50 values for the reference compounds were CX-4945 (pIC_50_ ≈ 9.0) and SGC-CK2-1 (pIC_50_ ≈ 8.38), respectively. Although the CX series and our identified scaffolds belong to distinct chemical classes, the pIC_50_ values of our selected top hits demonstrate consistency with experimentally reported ranges, and the comparable pIC_50_ profiles indicate that the newly proposed hits exhibit similar binding potency toward CK2. Notably, Anastatin B exhibited a predicted pIC_50_ comparable to CX-4945 and SGC-CK2-1, suggesting near-clinical level potency, while compounds such as 3,4,8,9,10-pentahydroxy-dibenzo-[b,d]pyran-6-one and Rhein demonstrated activity profiles consistent with selective chemical probes like SGC-CK2-1. This suggests that these compounds possess a strong inhibitory potential and can serve as promising starting points for structure-guided lead optimization to achieve enhanced activity and selectivity.

## 3. Materials and Methods

### 3.1. CK2 Structure Retrieval and Preparation

The crystallographic coordinates of Casein Kinase 2 (CK2) were retrieved from the protein databank (RCSB) using the accession number: 3NGA [27]. The CK2 kinase complex with CK2-CX-4945 was subjected to structure preparation and refinement. Using the protein preparation wizard in Schrodinger Maestro 2023, the complex was prepared. Any missing residue was modeled with the fill-in missing loop and side chains using the Prime tool [28]. The structure was preprocessed, and the protonation states were correctly defined. The structure was optimized by using PROPKA and setting the pH to 7.0. Then, the OPLS force field was used to minimize the complex.

### 3.2. Benchmarking and Validation of the Molecular Screening Protocol

A benchmark library was constructed by retrieving 534 experimentally validated actives from ChEMBL (accessed on 25 Januray, 2026) and related literature, which were standardized in RDKit (salt removal, tautomer/valence normalization, stereochemistry preservation) and converted to 3D with protonation at pH 7.0 and MMFF minimization [29,30]. To balance the dataset, 2922 property-matched but topologically distinct decoys were generated using a DUD-E workflow, ensuring similarity in MW, cLogP, HBD/HBA, rotatable bonds, and charge while avoiding scaffold overlap; these were likewise protonated, minimized, and deduplicated [31]. The receptor was prepared in Maestro with the Protein Preparation Wizard (bond orders, H-bond optimization, restrained OPLS minimization), and Glide Extra Precision (XP) docking was performed using grids centered on the crystallographic ligand, enabling conformational sampling and XP scoring [32]. Docking scores were oriented such that higher values consistently represented stronger predicted binding. Evaluation metrics included ROC–AUC, PR–AUC, enrichment factors (EF@1/2/5%), Top-N enrichment (50 and 384 ligands), and early recognition metrics (BEDROC, RIE at α = 20, 80.5, 160.9) with 500 bootstrap confidence intervals [33]. Visualization consisted of shaded ROC and PR curves, cumulative enrichment with early percentile markers, gradient EF bar plots, violin + jitter distributions of docking scores, and a PCA projection of ECFP4 fingerprints to explore chemical space diversity [34].

### 3.3. Exploring the Chemical Space for Novel Hits Identification

For the CK2, we prepared the compound library using a database management tool in Schrodinger Maestro [35]. We built a library of retrieved natural product libraries, such as the North African natural compounds database (NANDB), the East African natural compounds database (EANDB), and the coconut natural compounds databases [36,37]. The QikProp option was executed, and a prefilter by Lipinski’s Rule was applied to prepare and filter these databases for drug-like compounds that obey R5 rules. Each ligand was prepared by using the “ligprep” option in Schrodinger Maestro by using the Epik option and removing high-energy ionization/tautomer states. The 2D stereo properties were used to obtain stereochemical information, while 4 stereoisomers were retained. A single low-energy conformation was generated for each ligand.

### 3.4. Receptor Grid Generation and Molecular Screening

The prepared drug databases and CK2 receptor were used as input for the identification of potential hits in the chemical space of natural products. The active site was defined as the grid center: x = 1.505, y = −1.461, and −10.265. The inner box was defined as 10 × 10 × 10, while the outer box was defined as 25.33 × 25.33 × 25.33 each. A three-step approach, HTVS, SP, and XP docking, was applied for more accurate results. A flexible docking with a penalized non-planar conformation approach was used, while post-docking minimization was also performed, with 1 pose for each compound using the constraints for each complex. The top 10% were then selected for the second step. In the HTVS, all the states were retained, while in the SP approach, only good-scoring states were selected for the next step. From XP docking, top hits were selected by using the best-scoring hits option. The interactions of the top hits were visualized in PyMOL and Discovery Studio Visualizer [38,39].

### 3.5. Molecular Dynamics Simulation

The pharmacological potential and inhibitory mechanism of the top hits were explored through atomic simulation using the Amber 24 simulation software. In the first step, the ligand was parameterized, and for proteins, the “FF19SB” force field was used [40]. Then, we solvated each complex by adding a cubic box of TIP3P water model with a cut-off distance of 12 Å. For neutralization, counter ions, i.e., Na^+^ and Cl^−^, were supplied to the system [41]. To ensure the system was fit for optimal performance, gentle minimization of 12,000 and 5000 steps was performed by using the steepest descent and conjugate gradient algorithms. Then, each system was gradually heated from 0 to 325 (K) [42], and the hydrogen bonds were treated via the SHAKE method. The Langevin thermostat was used for temperature, while the barostat was used for maintaining a pressure of 1 atm and a temperature of 310 K [43]. Using the pressure and temperature, 10-nanosecond (ns) time equilibrations were achieved. Afterward, the MD simulation was conducted for 200 nanoseconds (ns) total time at a constant temperature of 325 K [44]. We further thoroughly examined the conformational alterations that eventuated during simulations. To analyze these alterations, we utilized the CPPTRAJ module within AMBER20 to investigate several factors, namely root mean square deviation (RMSD), root mean square fluctuation (RMSF), radius of gyration (RoG), and principal component analysis (PCA). The radius of gyration (RoG) is a valuable measure for analyzing protein size and compaction [45]. Finally, data visualization and analyses were performed with the help of the Origin Pro Software 2024b version.

### 3.6. Binding Free Energy Calculation

The binding free energy calculation is the most widely used approach to re-evaluate the accurate binding strength of a system [46]. Because of the broader applications, owing to advantages over other methods, i.e., computational efficiency and less time requirements, this approach has been widely implemented. For each complex, the van der Waals (vdW), electrostatic, solvent-accessible surface area (SASA), as well as the generalized Born (GB) components, were determined based on the entire simulation trajectory. This method merges molecular mechanics simulations, which describe the interactions between atoms, with implicit solvent models, which describe the interactions between the protein and solvent, to compute the binding strength [47,48,49]. Hence, we also applied this approach here to accurately compute the total binding free energy of the protein–ligand complexes. Mathematically, the binding free energy can be estimated as:

(1) “ΔG(bind) = ΔG(complex) − [ΔG(receptor) + ΔG(ligand)]”

Different contributing components of total binding energy were calculated by the following equation:

(2) “G=Gbond + Gele +GvdW +Gpol +Gnpol”

### 3.7. Frontier Molecular Orbital (FMO) and Electronic Structure Analysis

The electronic structure calculations were carried out using a custom HOMO–LUMO computational pipeline developed in Python, integrating RDKit for molecular preprocessing, PySCF for quantum chemical calculations, and PyMOL for orbital visualization [50,51]. Molecular structures were first generated from SMILES representations using RDKit. Hydrogen atoms were added explicitly, and the three-dimensional geometries were embedded using the ETKDGv3 algorithm followed by optimization with the Universal Force Field (UFF). These optimized geometries were converted into Cartesian coordinates (XYZ format) and served as input for quantum chemical calculations. All electronic structure computations were performed with PySCF (Python for Strongly Correlated Electron Systems). The Density Functional Theory (DFT) method was employed using the PBE0 functional with the 6-31G* basis set. For closed-shell molecules, restricted Hartree–Fock (RHF) or restricted Kohn–Sham (RKS) formalisms were applied, while restricted open-shell Hartree–Fock (ROHF) was used where required. Convergence criteria were set with a tolerance of 1 × 10^−8^ Hartree to ensure high accuracy. The frontier molecular orbitals (FMOs) were determined from the computed molecular orbital energy spectrum. The highest occupied molecular orbital (HOMO) and the lowest unoccupied molecular orbital (LUMO) indices were identified based on electron count, and their orbital energies were extracted. The energy gap (ΔE = E_LUMO_ − E_HOMO_) was calculated in electronvolts (eV) using a Hartree-to-eV conversion factor of 27.2114. Additionally, the dipole moment vector and magnitude were calculated to provide insights into the polarity and electronic distribution of each molecule. HOMO and LUMO orbital density maps were exported as cube files, and PyMOL scripts were automatically generated for high-resolution orbital visualization.

### 3.8. Machine Learning-Driven QSAR Modeling

We developed a comprehensive, AI-driven QSAR modeling pipeline to systematically evaluate CK2 inhibition (CHEMBL3629) using multi-level molecular representations and state-of-the-art machine learning. Bioactivity data were retrieved from the ChEMBL database under stringent filtering criteria (IC_50_ in nM, equality relations only), standardized to canonical SMILES, and rigorously curated to remove invalid or extreme values prior to transformation into pIC_50_ (−log_10_(IC_50_[M])) within a defined dynamic range of 3–12. Structural information was enriched by computing an extensive suite of physicochemical and topological 2D descriptors (including LogP, TPSA, Estate VSA, SMR VSA, PEOE VSA, Kier–Hall indices, and Chi connectivity indices), alongside geometry-refined 3D shape descriptors (asphericity, eccentricity, PMI1–3, radius of gyration, and spherocity index) generated from MMFF-optimized ETKDG conformers with fixed random seeds to ensure reproducibility. Complementarily, six orthogonal fingerprinting schemes (ECFP4/6, FCFP4/6, MACCS, RDKit) were computed using MolFeat to capture circular, pharmacophoric, and substructure-level features, followed by low-variance filtering (threshold = 0.001) and median imputation of missing values. Feature representations were assembled into modular spaces (2D, 3D, FP, and combined), enabling systematic evaluation of descriptor contributions. To mitigate descriptor redundancy and high dimensionality, feature selection was performed using an embedded tree-based approach (ExtraTrees) within the modeling pipeline. A Bemis–Murcko scaffold-based splitting strategy was applied to construct an external test set, ensuring structural independence between training and test compounds and preventing chemical series leakage. Within the remaining data, stratified sampling based on pIC_50_ distribution (five bins) was used for training–validation partitioning. A diverse panel of machine learning algorithms was evaluated, including linear models (Ridge, ElasticNet, Partial Least Squares), kernel-based methods (SVR), ensemble learners (Random Forest, ExtraTrees, Gradient Boosting, HistGradientBoosting), neural networks (MLP with 100–50 hidden layers), and GPU-accelerated boosting frameworks (XGBoost, LightGBM). Hyperparameters for key models were optimized using Optuna (30 Bayesian-guided trials with cross-validation), ensuring an appropriate bias–variance trade-off. All preprocessing steps, including imputation, scaling, and feature selection, were implemented within scikit-learn pipelines, ensuring transformations were confined to training folds and eliminating data leakage. To extend beyond descriptor-based representations, a Graph Isomorphism Network (GIN) was implemented using PyTorch Geometric with atom-level features, dual GINConv layers (hidden dimension = 64, ReLU activation), global add pooling, and early stopping (Adam optimizer, learning rate = 1 × 10^−3^, batch size = 64, patience = 8, maximum 60 epochs) to prevent overfitting. Ensemble learning was further explored through stacking, combining top-performing base learners with a Ridge regression meta-model to enhance predictive stability. Model performance was evaluated using R^2^, RMSE, and MAE across both cross-validation and an external scaffold-based test set, with overfitting monitored via ΔR^2^. Model robustness was further validated using Y-scrambling, which yielded near-zero predictive performance for randomized targets, confirming the absence of chance correlations. Applicability domain (AD) analysis was performed using k-nearest neighbor distance metrics to ensure predictions were confined to reliable chemical space. Prediction uncertainty was additionally quantified using conformal prediction (MAPIE), providing 90% prediction intervals and coverage estimates. Reproducibility was ensured by fixing random seeds (SEED = 42), logging software environments, enabling GPU acceleration where available, and archiving descriptors, feature matrices, and model outputs in machine-readable formats.

Stacking ensembles were constructed separately for each feature space using the *StackingRegressor* framework implemented in scikit-learn. After training the individual base regressors on the preprocessed training data, models with positive external test set performance were retained, and the top three base learners within each feature set were selected according to their test R^2^ values. These selected regressors were then combined into a two-level ensemble, where their predictions served as inputs to a Ridge regression meta-learner (alpha = 1.0). Before stacking, all input features were standardized using *StandardScaler*, and for high-dimensional feature spaces (>100 variables), embedded feature selection was performed using *SelectFromModel* with an *ExtraTreesRegressor* and a median importance threshold. The stacking model was trained on the scaled/selected training matrix and evaluated on the independent test set using R^2^, RMSE, and MAE. In the revised analysis, cross-validation performance for stacking ensembles was additionally computed using 5-fold KFold cross-validation on the same transformed training matrix, and the corresponding CV R^2^ mean and standard deviations were calculated. Collectively, this pipeline integrates classical QSAR rigor with modern machine learning and graph-based approaches, establishing a robust and reproducible framework for kinase inhibitor prediction under realistic chemical space constraints. [52,53,54].

### 3.9. Hardware and Reproducibility

All computations were performed on Linux with Python 3.10. Reproducibility was ensured by fixing random seeds (42) and recording all software versions. Where available, GPU acceleration (NVIDIA CUDA 12.4) was employed using RTX4090 with i9-13900K. Outputs, including feature matrices, trained models, and performance metrics, were saved in JSON and CSV formats for transparency.

## 4. Conclusions

This study delivers a comprehensive AI-guided discovery platform uniting data-driven learning with atomistic simulations to advance CK2-targeted therapy in TNBC. Through systematic benchmarking, several natural-product-derived scaffolds, particularly Anastatin B and aloe emodin acetate, demonstrated superior binding stability, dynamic compactness, and favorable quantum descriptors compared with the clinical reference CX-4945. The convergence of machine learning predictions, molecular dynamics validation, and quantum-mechanical energy profiling underscores the translational value of integrative computational pipelines in anti-cancer drug discovery. Despite the encouraging results, several limitations should be acknowledged. Model performance depends on the quality and diversity of experimental data, which may limit reliability for novel chemical space. Variability in bioactivity data can introduce uncertainty, and the use of multiple descriptors may still lead to redundancy and overfitting. Additionally, docking and MM-GBSA provide approximate binding estimates and do not fully capture protein flexibility or biological complexity. Future work should focus on larger high-quality datasets and experimental validation. Future optimization of these leads through fragment-growth and free energy perturbation approaches could yield next-generation CK2 inhibitors with enhanced potency and selectivity.

## Figures and Tables

**Table 1:** Extra precision-based predicted top leads from natural product chemical space. The docking scores are given in kcal/mol. The table shows the 2D structures of the selected top hits and the control (CX-4945) ranked using docking scores. The interacting residues involved in hydrogen bonding, hydrophobic, and other interactions are also mentioned.

S. No	2D Structure	Name	Hydrogen Bonding Residues	Hydrophobic/Other Interactions	Docking Scores
**1.**	** 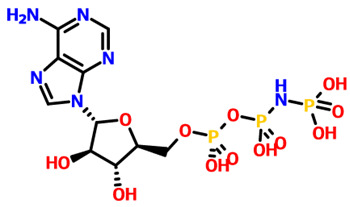 **	CX-4945	Arg47, Lys49, Tyr50, Lys68, Ser51, Val116		**−9.57**
**2.**	** 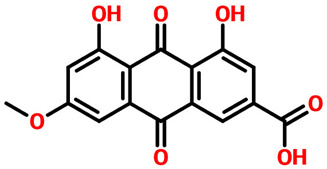 **	Parietinic Acid	Lys68, Glu114 and Val116	Leu45, Val53, Val66, Lys68, Ile95, Phe113, Met163 and Ile174	**−11.65**
**3.**	** 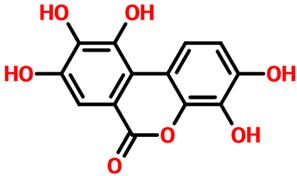 **	Rhein	Lys68, Glu114, Val116 and Asp175	Val53, Val66, Lys68, Ile95, Met163 and Ile174	**−12.36**
**4.**	** 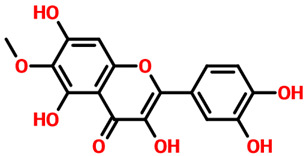 **	6-methoxy quercetin	Leu45, Lys68, Glu114, Val116 and Asp175	Val53, Val66, Lys68, Ile95, Met163 and Ile174	**−11.60**
**5.**	** 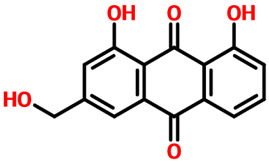 **	3,4,8,9,10-pentahydroxy-dibenzo-[b,d]pyran-6-one	Lys68, Val116 and Asp175	Gly46, Val53, Val66, Ile95, Met163 and Ile174	**−12.36**
**6.**	** 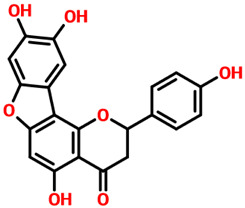 **	Anastatin B	Arg47, Lys68, Val116 and Asp175	Gly46, Val53, Val66, Ile95, Met163 and Ile174	**−13.12**
**7.**	** 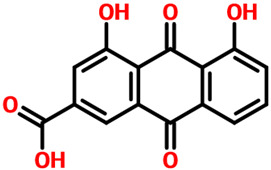 **	Aloe Emodin	Glu114, Val116, Asn118 and Asp175	Leu45, Val53, Val66, Lys68, Ile95, Phe113, Met163 and Ile174	**−11.73**

**Table 2:** MM-PBSA binding free energy decomposition of the control and selected top-hit complexes calculated over different simulation intervals (1–10 ns, 11–30 ns, and 186–200 ns). All energy values are reported in kcal/mol as mean ± standard error.

	MM/PBSA	Control	Anastatin B	Aloe Emodin	Parietinic Acid	3–4 Penta	6-MQ	Rhein
**1–10 ns**	**vdWaals**	−33.58 ± 0.15	−42.78 ± 0.32	−39.02 ± 0.13	−29.14 ± 0.11	−24.99 ± 0.19	−38.41 ± 0.15	−27.27 ± 0.29
	**EEL**	−21.62 ± 0.31	−9.12 ± 0.21	−5.97 ± 0.21	−122.47 ± 0.53	−9.12 ± 0.18	−13.47 ± 0.20	−175.31 ± 1.46
	**EPB**	42.56 ± 0.40	28.53 ± 0.29	21.50 ± 0.19	137.44 ± 0.51	20.23 ± 0.28	31.00 ± 0.20	190.65 ± 1.11
	**ENPOLAR**	−2.87 ± 0.00	−3.46 ± 0.29	−3.59 ± 0.00	−3.11 ± 0.07	−2.48 ± 0.13	−3.07 ± 0.06	−2.86 ± 0.011
	**EDSPIDER**	0.00 ± 0.00	0.00 ± 0.00	0.00 ± 0.00	0.00 ± 0.00	0.00 ± 0.00	0.00 ± 0.00	0.00 ± 0.00
	**Delta G Gas**	−55.21 ± 0.42	−51.91 ± 0.26	−44.99 ± 0.28	−151.62 ± 0.55	−34.12 ± 0.29	−51.89 ± 0.26	−202.61 ± 1.26
	**Delta G Solv**	39.68 ± 0.40	25.06 ± 0.28	17.90 ± 0.19	134.33 ± 0.51	17.75 ± 0.27	27.93 ± 0.20	187.78 ± 1.12
	**Delta Total**	**−15.52 ± 0.16**	**−26.85 ± 0.20**	**−27.09 ± 0.22**	**−17.28 ± 0.10**	**−16.37 ± 0.15**	**−23.95 ± 0.18**	**−14.82 ± 0.37**
**11–30 ns**	**vdWaals**	−34.85 ± 0.13	−43.68 ± 0.14	−38.83 ± 0.08	−30.65 ± 0.10	−21.21 ± 0.18	−37.02 ± 0.12	−24.19 ± 0.15
	**EEL**	−24.13 ± 0.25	−8.22 ± 0.10	−4.51 ± 0.12	−125.01 ± 0.61	−7.9 ± 0.12	−12.83 ± 0.11	−169.14 ± 1.13
	**EPB**	45.71 ± 0.32	28.04 ± 0.14	20.29 ± 0.11	141.30 ± 0.55	17.11 ± 0.19	29.75 ± 0.15	181.00 ± 1.06
	**ENPOLAR**	−2.86 ± 0.00	−3.45 ± 0.00	−3.57 ± 0.00	−3.22 ± 0.00	−2.16 ± 0.01	−3.05 ± 0.04	−2.67 ± 0.00
	**EDSPIDER**	0.00 ± 0.00	0.00 ± 0.00	0.00 ± 0.00	0.00 ± 0.00	0.00 ± 0.00	0.00 ± 0.00	0.00 ± 0.00
	**Delta G Gas**	−58.99 ± 0.35	−51.91 ± 0.15	−43.34 ± 0.17	−155.66 ± 0.59	−29.12 ± 0.26	−49.85 ± 0.17	−193.35 ± 1.09
	**Delta G Solv**	42.85 ± 0.12	24.59 ± 0.14	16.72 ± 0.11	138.08 ± 0.55	14.95 ± 0.18	26.69 ± 0.15	178.33 ± 1.06
	**Delta Total**	**−16.14 ± 0.12**	**−27.32 ± 0.10**	**−26.62 ± 0.13**	**−17.58 ± 0.13**	**−14.17 ± 0.12**	**−23.16 ± 0.10**	**−15.01 ± 0.16**
**186–200 ns**	**vdWaals**	−32.45 ± 0.10	−43.89 ± 0.09	−36.42 ± 0.06	−32.43 ± 0.9	−24.05 ± 0.07	−31.57 ± 0.06	−13.59 ± 0.17
	**EEL**	−19.14 ± 0.16	−9.14 ± 0.09	−7.13 ± 0.07	−160.87 ± 0.71	−13.64 ± 0.18	−2.69 ± 0.07	−205.86 ± 1.43
	**EPB**	37.88 ± 0.23	28.63 ± 0.12	22.94 ± 0.09	174.16 ± 0.65	25.90 ± 0.22	18.14 ± 0.09	206.52 ± 1.44
	**ENPOLAR**	−2.80 ± 0.00	−3.44 ± 0.12	−3.52 ± 0.00	−3.19 ± 0.00	−2.36 ± 0.03	−2.95 ± 0.02	−1.58 ± 0.01
	**EDSPIDER**	0.00 ± 0.00	0.00 ± 0.00	0.00 ± 0.00	0.00 ± 0.00	0.00 ± 0.00	0.00 ± 0.00	0.00 ± 0.00
	**Delta G Gas**	−51.59 ± 0.23	−53.04 ± 0.14	−43.56 ± 0.10	−193.31 ± 0.68	−37.69 ± 0.22	−34.26 ± 0.09	−219.47 ± 1.49
	**Delta G Solv**	35.08 ± 0.23	25.84 ± 0.08	19.42 ± 0.09	170.97 ± 0.65	23.54 ± 0.22	15.19 ± 0.08	204.94 ± 1.43
	**Delta Total**	**−16.51 ± 0.08**	**−27.84 ± 0.08**	**−24.13 ± 0.08**	**−22.34 ± 0.11**	**−14.15 ± 0.07**	**−19.07 ± 0.07**	**−14.53 ± 0.14**

**Table 3:** MM-GBSA binding free energy decomposition of the control and selected top-hit complexes calculated over different simulation intervals (1–10 ns, 11–30 ns, and 186–200 ns). All energy values are reported in kcal/mol as mean ± standard error.

	MM/GBSA	Control	Anastatin B	Aloe Emodin	Parietinic Acid	3–4 Penta	6-MQ	Rhein
**1–10 ns**	**vdWaals**	−33.58 ± 0.15	−42.78 ± 0.32	−39.02 ± 0.13	−29.14 ± 0.11	−24.99 ± 0.19	−38.41 ± 0.15	−27.27 ± 0.29
	**EEL**	−21.62 ± 0.31	−9.12 ± 0.21	−5.97 ± 0.21	−122.47 ± 0.53	−9.12 ± 0.18	−13.47 ± 0.20	−175.31 ± 1.46
	**EGB**	43.05 ± 0.29	27.53 ± 0.29	16.82 ± 0.19	139.67 ± 0.51	24.35 ± 0.19	32.74 ± 0.16	189.07 ± 1.29
	**ESURF**	−4.13 ± 0.01	−4.92 ± 0.25	−5.19 ± 0.01	−4.20 ± 0.07	−3.14 ± 0.13	−4.71 ± 0.12	−3.91 ± 0.03
	**Delta G Gas**	−55.21 ± 0.42	−51.91 ± 0.26	−44.99 ± 0.28	−151.62 ± 0.55	−34.12 ± 0.29	−51.89 ± 0.26	−202.61 ± 1.26
	**Delta G Solv**	39.68 ± 0.40	22.67 ± 0.15	11.64 ± 0.15	135.15 ± 0.51	21.20 ± 0.27	28.02 ± 0.16	185.16 ± 1.31
	**Delta Total**	**−16.29 ± 0.16**	**−29.23 ± 0.20**	**−33.36 ± 0.17**	**−16.15 ± 0.89**	**−12.91 ± 0.15**	**−23.86 ± 0.17**	**−17.44 ± 0.66**
**11–30 ns**	**vdWaals**	−34.85 ± 0.13	−43.68 ± 0.14	−38.83 ± 0.08	−30.65 ± 0.10	−21.21 ± 0.18	−37.02 ± 0.12	−24.19 ± 0.15
	**EEL**	−24.13 ± 0.25	−8.22 ± 0.10	−4.51 ± 0.12	−125.01 ± 0.61	−7.9 ± 0.12	−12.83 ± 0.11	−169.14 ± 1.32
	**EGB**	45.71 ± 0.32	27.08 ± 0.14	15.82 ± 0.92	143.20 ± 0.56	21.41 ± 0.19	31.50 ± 0.10	181.31 ± 1.04
	**ESURF**	−4.12 ± 0.08	−4.96 ± 0.01	−5.17 ± 0.07	−4.43 ± 0.01	−2.69 ± 0.01	−4.54 ± 0.13	−3.46 ± 0.18
	**Delta G Gas**	−58.99 ± 0.35	−51.91 ± 0.15	−43.34 ± 0.17	−155.66 ± 0.59	−29.12 ± 0.26	−49.85 ± 0.17	−193.35 ± 1.09
	**Delta G Solv**	41.14 ± 0.23	22.11 ± 0.09	10.65 ± 0.88	138.77 ± 0.56	18.71 ± 0.15	22.96 ± 0.10	177.85 ± 1.04
	**Delta Total**	**−17.85 ± 0.14**	**−27.79 ± 0.09**	**−32.69 ± 0.11**	**−16.89 ± 0.97**	**−10.40 ± 0.12**	**−22.89 ± 0.11**	**−15.49 ± 0.12**
**186–200 ns**	**vdWaals**	−32.45 ± 0.10	−43.89 ± 0.09	−36.42 ± 0.06	−32.43 ± 0.9	−24.05 ± 0.07	−31.57 ± 0.06	−13.59 ± 0.17
	**EEL**	−19.14 ± 0.16	−9.14 ± 0.09	−7.13 ± 0.07	−160.87 ± 0.71	−13.64 ± 0.18	−2.69 ± 0.07	−205.86 ± 1.43
	**EGB**	38.14 ± 0.17	28.02 ± 0.12	20.91 ± 0.65	174.92 ± 0.64	29.12 ± 0.22	20.09 ± 0.65	208.16 ± 1.44
	**ESURF**	−3.79 ± 0.00	−4.96 ± 0.12	−4.83 ± 0.08	−4.39 ± 0.08	−3.11 ± 0.06	−4.25 ± 0.06	−1.83 ± 0.01
	**Delta G Gas**	−51.59 ± 0.23	−53.04 ± 0.14	−43.56 ± 0.10	−193.31 ± 0.68	−37.69 ± 0.22	−34.26 ± 0.09	−219.47 ± 1.49
	**Delta G Solv**	34.34 ± 0.17	23.06 ± 0.08	16.08 ± 0.69	170.52 ± 0.65	26.01 ± 0.19	15.83 ± 0.63	206.32 ± 1.43
	**Delta Total**	**−17.25 ± 0.08**	**−27.97 ± 0.07**	**−27.47 ± 0.07**	**−22.78 ± 0.91**	**−11.68 ± 0.63**	**−18.42 ± 0.05**	**−13.15 ± 0.15**

**Table 4:** Summary of model performance metrics for different feature sets. The table reports the coefficient of determination (R^2^) for training, test, and cross-validation (CV) datasets, along with the difference between training and test performance (ΔR^2^). Values are presented as mean ± standard deviation (SD) across cross-validation folds.

Feature Set	Model	Train R^2^	Test R^2^	ΔR^2^	CV R^2^ (±SD)
**2D**	**Random Forest**	0.800	0.652	0.148	0.633 ± 0.036
	**Gradient Boosting**	0.807	0.653	0.155	0.639 ± 0.044
	**HistGB**	0.876	0.642	0.235	0.624 ± 0.064
	**ExtraTrees**	0.884	0.644	0.240	0.620 ± 0.051
	**SVM**	0.803	0.603	0.200	0.626 ± 0.024
	**KNN**	0.725	0.555	0.170	0.546 ± 0.055
	**Stacking**	0.854	0.672	0.182	0.664 ± 0.044
**3D**	**Random Forest**	0.746	0.459	0.287	0.460 ± 0.051
	**Extra Trees**	0.884	0.543	0.342	0.445 ± 0.079
	**Stacking**	0.835	0.536	0.299	0.530 ± 0.034
**FP**	**Random Forest**	0.799	0.668	0.130	0.666 ± 0.041
	**Gradient Boosting**	0.803	0.664	0.139	0.664 ± 0.043
	**HistGB**	0.875	0.680	0.195	0.656 ± 0.063
	**ElasticNet**	0.801	0.675	0.126	0.656 ± 0.036
	**Stacking**	0.841	0.690	0.151	0.693 ± 0.0036
**2D + 3D**	**Random Forest**	0.853	0.650	0.202	0.628 ± 0.046
	**Gradient Boosting**	0.806	0.647	0.159	0.636 ± 0.047
	**Stacking**	0.858	0.672	0.186	0.669 ± 0.046
**2D + FP**	**Random Forest**	0.840	0.672	0.168	0.661 ± 0.042
	**Gradient Boosting**	0.818	0.688	0.130	0.674 ± 0.035
	**Stacking**	0.840	0.691	0.150	0.689 ± 0.038
**3D + FP**	**Random Forest**	0.844	0.673	0.171	0.663 ± 0.042
	**Gradient Boosting**	0.812	0.668	0.144	0.669 ± 0.038
	**Stacking**	0.845	0.688	0.157	0.694 ± 0.039
**Combined**	**Random Forest**	0.817	0.681	0.136	0.667 ± 0.040
	**Gradient Boosting**	0.823	0.684	0.139	0.694 ± 0.039
	**Stacking**	0.851	0.692	0.160	0.690 ± 0.039
**GNN**	**GIN**	0.040	0.020	0.020	−

## Data Availability

The original contributions presented in this study are included in the article/Supplementary Material. Further inquiries can be directed to the corresponding authors.

## Supplementary Materials

The following supporting information can be downloaded at: https://www.mdpi.com/article/10.3390/ph19050694/s1, Figure S1: HOMO–LUMO energy profile and frontier molecular orbital (FMO) visualization of top CK2 inhibitors; Figure S2: Frontier molecular orbitals of selected CK2 inhibitors showing electron density distribution; Table S1: Performance of machine learning models across descriptor and fingerprint feature sets for CK2 inhibitor prediction.

## Abbreviations

WHO	World Health Organization,
TNBC	triple-negative breast cancer,
HER2	human epidermal growth factor receptor 2,
ER	estrogen receptor,
PR	progesterone receptor,
ROC–AUC	Receiver Operating Characteristic–Area Under the Curve
DFT	Density Functional Theory
RMSD	Root Mean Square Deviation
RMSF	Root Mean Square Fluctuation
PCA	Principal Component Analysis
Rg	Radius of Gyration
AMBER	Assisted Model Building with Energy Refinement
MM-GBSA	Molecular Mechanics Generalized Born Surface Area
MM-PBSA	Molecular Mechanics Poisson–Boltzmann Surface Area
HOMO–LUMO	Highest Occupied Molecular Orbital–Lowest Unoccupied Molecular Orbital
