# Supplementary material for: An Integrative Computational Pipeline for CK2 Inhibitor Discovery in Triple-Negative Breast Cancer Using Virtual Screening, Molecular Dynamics, Machine Learning, and Density Functional Theory
Source: Pharmaceuticals (Basel). 2026 Apr 28;19(5):694. doi: 10.3390/ph19050694 (PMC13209216; doi:10.3390/ph19050694)
Supplement: Supplementary file 1 [file pharmaceuticals-19-00694-s001.zip › pharmaceuticals-4234575-supplementary.pdf]

Supplementary Materials

An Integrative Computational Pipeline for CK2 Inhibitor Discovery in Triple-Negative Breast Cancer Using Virtual Screening, Molecular Dynamics, Machine Learning, and Density Functional Theory

Abbas Khan[1,2], Fahad M. Alshabrmi[3], Anwar Mohammad[4], Mohanad Shkoor[5], Eid A. Alatawi[6], Raed M. Al-Zoubi[7,8,9], Long Chiau Ming[2], Abdelali Agouni[1*]

1. Department of Pharmaceutical Sciences, College of Pharmacy, QU Health, Qatar University, P.O. Box 2713, Doha, Qatar. abbas.khan@qu.edu.qa /aagouni@qu.edu.qa
2. Division of Bioinformatics, Department of Biomedical Sciences, Faculty of Medical and Life Sciences, Sunway University, Bandar Sunway, Malaysia. chiaumingl@sunway.edu.my
3. Department of Medical Laboratories, College of Applied Medical Sciences, Qassim University, Buraydah 51452, Saudi Arabia.
4. Precision Health Analysis Unit, Translational Research, Dasman Diabetes Institute, Dasman, Kuwait. anwar.mohammad@dasman.institute.org
5. Department of Chemistry, College of Arts and Science, Qatar University, P.O. Box 2713, Doha, Qatar. mshkoor@qu.edu.qa
6. Department of Medical Laboratory Technology, Faculty of Applied Medical Sciences, University of Tabuk, Tabuk 71491, Saudi Arabia. eid.alatawi@ut.edu.sa
7. Surgical Research Section, Department of Surgery, Hamad Medical Corporation, Doha, Qatar. ralzoubi@hamad.qa
8. Department of Biomedical Sciences, College of Health Sciences, QU Health, Qatar University, P.O. Box 2713, Doha, Qatar.
9. Department of Chemistry, Jordan University of Science and Technology, P.O. Box 3030, Irbid, 22110, Jordan

[*]Correspondence should be sent to: Prof. Abdelali Agouni / Dr. Raed M. Al-Zoubi, Department of Pharmaceutical Sciences, College of Pharmacy, Qatar University, P.O. Box 2713, Doha, Qatar. Tel: +974 4403 5610; Email: aagouni@qu.edu.qa / Surgical Research Section, Department of Surgery, Hamad Medical Corporation, Doha, Qatar. ralzoubi@hamad.qa

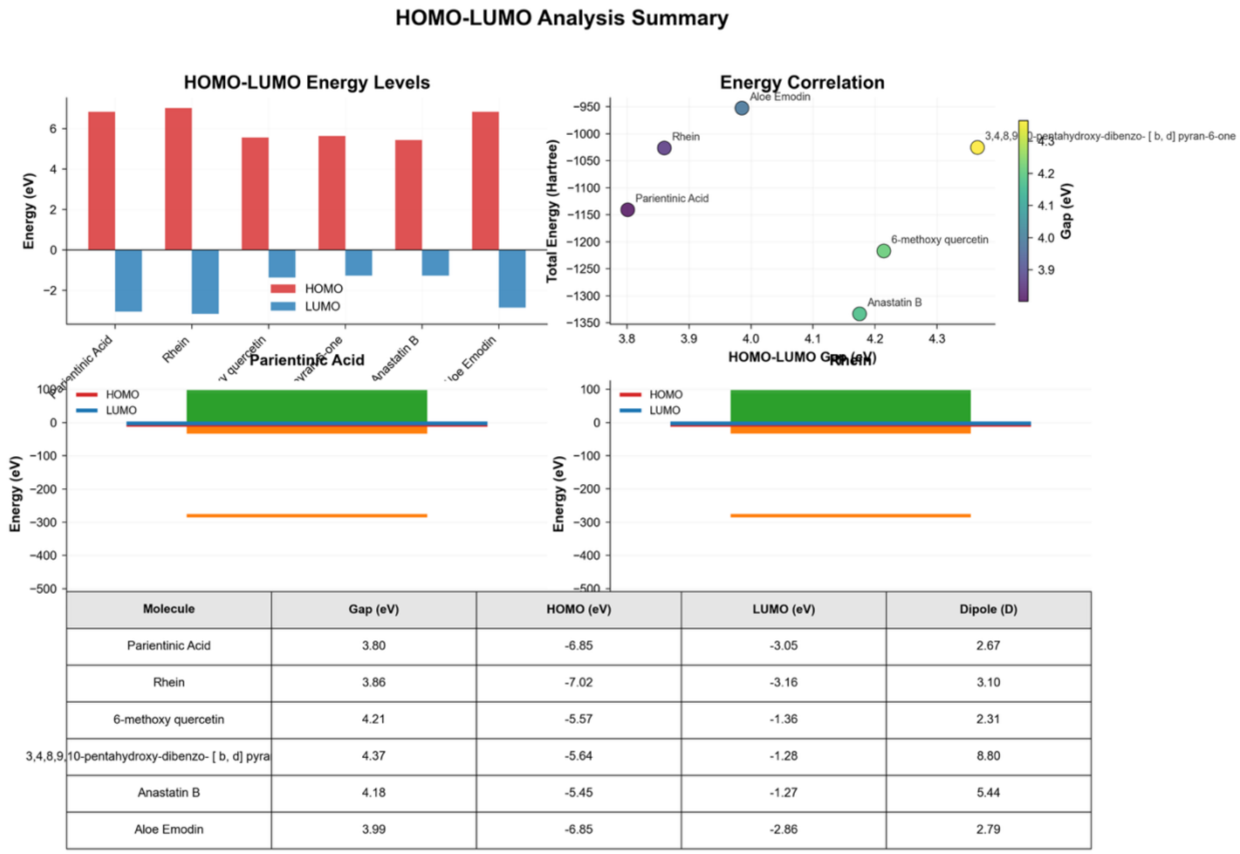

Molecule	Gap (eV)	HOMO (eV)	LUMO (eV)	Dipole (D)
Parientinic Acid	3.80	-6.85	-3.05	2.67
Rhein	3.86	-7.02	-3.16	3.10
6-methoxy quercetin	4.21	-5.57	-1.36	2.31
3,4,8,9,10-pentahydroxy-dibenzo- [b, d] pyra	4.37	-5.64	-1.28	8.80
Anastatin B	4.18	-5.45	-1.27	5.44
Aloe Emodin	3.99	-6.85	-2.86	2.79

Supplementary Figure S1. *HOMO–LUMO energy profile and frontier molecular orbital (FMO) visualization of top CK2 inhibitors.* (Top) Comparative quantum chemical analysis illustrating HOMO–LUMO energy levels, energy correlations, and electronic properties of the selected CK2 inhibitors. Parietinic acid and Rhein exhibited lower total energies and smaller HOMO–LUMO gaps (3.80–3.86 eV), indicating higher electronic reactivity, while 6-methoxyquercetin and Anastatin B displayed larger gaps (4.18–4.21 eV) consistent with greater kinetic stability. Dipole moment analysis further revealed that 3,4,8,9,10-pentahydroxy-dibenzo-[b,d]pyran-6-one possessed the highest polarity (8.80 D), suggesting enhanced intermolecular interaction potential.

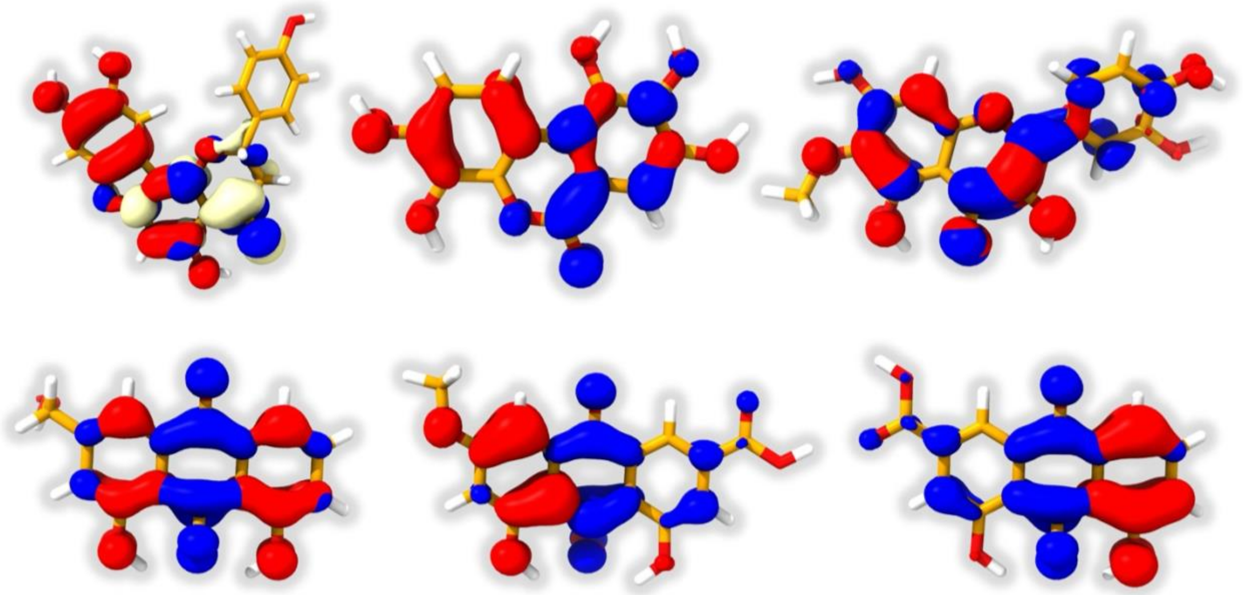

Supplementary Figure S2: *Frontier molecular orbitals of selected CK2 inhibitors showing electron density distribution.* HOMO (red) and LUMO (blue) surfaces depict the charge localization patterns responsible for molecular reactivity and potential interaction sites with CK2. The localized HOMO density on aromatic or oxygen-rich moieties indicates potential electron donation regions, while LUMO localization over carbonyl and hydroxyl sites suggests favorable electrophilic interaction regions contributing to binding affinity and reactivity.

Table S1. Performance of machine learning models across descriptor and fingerprint feature sets for CK2 inhibitor prediction. Values are reported to three decimal places. ΔR² represents the train–test generalization gap. CV metrics are shown as mean and standard deviation across folds.

Feature Set	Model	Train R^2	Test R^2	ΔR^2	CV R^2 Mean	CV R^2 SD	Train RMSE	Test RMSE	CV RMSE Mean	CV RMSE SD	Train MAE	Test MAE
**2D**	RandomForest	0.854	0.647	0.207	0.625	0.048	0.449	0.687	0.717	0.041	0.280	0.453
**2D**	XGBoost	0.846	0.657	0.188	0.644	0.043	0.462	0.677	0.700	0.037	0.311	0.469
**2D**	LightGBM	0.820	0.620	0.201	0.640	0.046	0.499	0.713	0.703	0.037	0.348	0.498
**2D**	GradientBoosting	0.807	0.653	0.155	0.639	0.044	0.516	0.682	0.704	0.034	0.369	0.485
**2D**	HistGB	0.877	0.645	0.231	0.622	0.065	0.413	0.689	0.720	0.053	0.227	0.416
**2D**	ExtraTrees	0.884	0.641	0.243	0.621	0.052	0.401	0.693	0.721	0.041	0.182	0.396
**2D**	SVM	0.765	0.604	0.161	0.619	0.022	0.570	0.728	0.725	0.022	0.376	0.495
**2D**	KNN	0.725	0.555	0.170	0.546	0.055	0.617	0.772	0.790	0.045	0.428	0.549
**2D**	MLP	0.860	-6025408702.829	6025408703.688	0.451	0.082	0.441	89797.423	0.866	0.045	0.279	5455.303
**2D**	ElasticNet	0.552	-211475000.294	211475000.845	0.482	0.056	0.787	16822.872	0.844	0.035	0.613	1022.541
**2D**	PLS	0.427	-38374251.789	38374252.215	-0.466	1.775	0.890	7166.227	1.235	0.677	0.703	436.013
**2D**	Ridge	0.584	-115248671.305	115248671.889	0.246	0.534	0.759	12419.056	0.968	0.295	0.588	755.027
**2D**	Stacking	0.837	0.662	0.175	0.671	0.041	0.475	0.673	0.668	0.053	0.325	0.466
**3D**	RandomForest	0.761	0.457	0.305	0.480	0.064	0.575	0.853	0.846	0.070	0.416	0.615
**3D**	XGBoost	0.811	0.468	0.343	0.475	0.073	0.511	0.844	0.850	0.070	0.366	0.596
**3D**	LightGBM	0.715	0.414	0.301	0.453	0.060	0.628	0.886	0.868	0.058	0.461	0.656
**3D**	GradientBoosting	0.648	0.408	0.240	0.449	0.057	0.698	0.890	0.872	0.065	0.522	0.662
**3D**	HistGB	0.811	0.432	0.379	0.445	0.086	0.511	0.872	0.873	0.071	0.354	0.615
**3D**	ExtraTrees	0.884	0.541	0.343	0.466	0.093	0.400	0.783	0.856	0.083	0.180	0.439
**3D**	SVM	0.582	0.308	0.274	0.417	0.063	0.760	0.962	0.897	0.063	0.509	0.694
**3D**	KNN	0.588	0.324	0.265	0.344	0.073	0.755	0.951	0.951	0.074	0.555	0.712
**3D**	MLP	0.462	-0.555	1.017	0.379	0.063	0.863	1.442	0.926	0.072	0.649	0.800
**3D**	ElasticNet	0.185	0.000	0.185	0.160	0.091	1.061	1.157	1.076	0.069	0.837	0.887
**3D**	PLS	0.148	-0.009	0.157	0.142	0.061	1.085	1.162	1.088	0.052	0.853	0.884
**3D**	Ridge	0.183	0.002	0.181	0.159	0.091	1.063	1.155	1.077	0.069	0.839	0.887
**3D**	Stacking	0.843	0.534	0.308	0.532	0.028	0.466	0.789	0.799	0.053	0.315	0.532
**FP**	RandomForest	0.849	0.664	0.185	0.665	0.044	0.457	0.671	0.679	0.039	0.293	0.445
**FP**	XGBoost	0.854	0.672	0.182	0.671	0.039	0.450	0.663	0.673	0.033	0.304	0.453
**FP**	LightGBM	0.835	0.676	0.160	0.673	0.049	0.477	0.659	0.670	0.045	0.324	0.463
**FP**	GradientBoosting	0.807	0.675	0.132	0.670	0.040	0.516	0.660	0.674	0.033	0.377	0.489
**FP**	HistGB	0.875	0.673	0.202	0.664	0.058	0.416	0.661	0.678	0.050	0.236	0.412
**FP**	ExtraTrees	0.884	0.568	0.316	0.589	0.054	0.401	0.761	0.751	0.033	0.182	0.428
**FP**	SVM	0.793	0.594	0.199	0.568	0.050	0.535	0.737	0.771	0.046	0.428	0.552
**FP**	KNN	0.742	0.570	0.172	0.588	0.035	0.597	0.759	0.753	0.030	0.411	0.540
**FP**	MLP	0.873	0.574	0.299	0.468	0.072	0.419	0.755	0.855	0.063	0.228	0.440
**FP**	ElasticNet	0.805	0.668	0.137	0.659	0.034	0.519	0.666	0.684	0.022	0.371	0.482
**FP**	PLS	0.545	0.525	0.020	0.520	0.032	0.793	0.798	0.813	0.032	0.592	0.603
**FP**	Ridge	0.882	0.649	0.233	0.493	0.082	0.403	0.685	0.831	0.049	0.190	0.393
**FP**	Stacking	0.847	0.688	0.160	0.698	0.039	0.459	0.646	0.641	0.054	0.310	0.451

**2D+3D**	RandomForest	0.819	0.653	0.165	0.633	0.042	0.501	0.681	0.710	0.039	0.348	0.479
**2D+3D**	XGBoost	0.839	0.663	0.177	0.638	0.049	0.471	0.672	0.705	0.045	0.322	0.467
**2D+3D**	LightGBM	0.808	0.618	0.190	0.630	0.048	0.515	0.715	0.713	0.036	0.363	0.499
**2D+3D**	GradientBoosting	0.807	0.641	0.166	0.645	0.039	0.517	0.694	0.699	0.030	0.369	0.487
**2D+3D**	HistGB	0.878	0.646	0.232	0.625	0.067	0.411	0.689	0.716	0.054	0.224	0.414
**2D+3D**	ExtraTrees	0.884	0.649	0.236	0.618	0.055	0.400	0.686	0.724	0.042	0.180	0.389
**2D+3D**	SVM	0.784	0.600	0.184	0.615	0.020	0.546	0.731	0.729	0.025	0.423	0.536
**2D+3D**	KNN	0.722	0.541	0.180	0.549	0.047	0.621	0.783	0.788	0.039	0.429	0.556
**2D+3D**	MLP	0.867	-1215692526.997	1215692527.863	0.394	0.153	0.430	40335.040	0.906	0.101	0.259	2450.667
**2D+3D**	ElasticNet	0.577	0.460	0.117	0.490	0.070	0.765	0.850	0.837	0.046	0.591	0.639
**2D+3D**	PLS	0.418	-23323404.626	23323405.044	-0.212	1.234	0.897	5586.845	1.171	0.524	0.708	340.077
**2D+3D**	Ridge	0.595	-8012705.276	8012705.871	0.474	0.114	0.748	3274.615	0.846	0.076	0.577	199.547
**2D+3D**	Stacking	0.861	0.671	0.190	0.669	0.046	0.439	0.663	0.670	0.059	0.280	0.438
**2D+FP**	RandomForest	0.815	0.684	0.131	0.667	0.038	0.505	0.650	0.677	0.032	0.363	0.468
**2D+FP**	XGBoost	0.837	0.677	0.160	0.666	0.035	0.475	0.658	0.678	0.031	0.335	0.462
**2D+FP**	LightGBM	0.838	0.680	0.158	0.662	0.043	0.474	0.655	0.681	0.033	0.318	0.445
**2D+FP**	GradientBoosting	0.821	0.686	0.135	0.673	0.042	0.498	0.649	0.670	0.037	0.359	0.464
**2D+FP**	HistGB	0.879	0.665	0.214	0.642	0.053	0.410	0.669	0.701	0.043	0.219	0.403
**2D+FP**	ExtraTrees	0.884	0.575	0.309	0.601	0.052	0.401	0.754	0.740	0.031	0.182	0.422
**2D+FP**	SVM	0.785	0.583	0.202	0.559	0.055	0.545	0.747	0.779	0.048	0.441	0.567
**2D+FP**	KNN	0.739	0.563	0.177	0.588	0.028	0.600	0.765	0.754	0.029	0.408	0.544
**2D+FP**	MLP	0.878	-155467041.799	155467042.677	0.505	0.107	0.411	14424.135	0.820	0.078	0.221	876.639
**2D+FP**	ElasticNet	0.820	-1684278.596	1684279.416	0.649	0.042	0.499	1501.335	0.694	0.027	0.349	91.661
**2D+FP**	PLS	0.540	-1144.413	1144.953	0.516	0.031	0.798	39.152	0.817	0.033	0.597	2.978
**2D+FP**	Ridge	0.883	-1146.271	1147.154	0.480	0.095	0.402	39.184	0.842	0.059	0.189	2.780
**2D+FP**	Stacking	0.829	0.692	0.137	0.687	0.036	0.486	0.642	0.652	0.052	0.344	0.455
**3D+FP**	RandomForest	0.842	0.679	0.163	0.671	0.042	0.468	0.656	0.672	0.036	0.319	0.461
**3D+FP**	XGBoost	0.852	0.667	0.186	0.668	0.042	0.452	0.668	0.676	0.038	0.309	0.458
**3D+FP**	LightGBM	0.841	0.673	0.168	0.665	0.053	0.469	0.662	0.678	0.048	0.315	0.458
**3D+FP**	GradientBoosting	0.814	0.669	0.145	0.666	0.041	0.508	0.666	0.678	0.039	0.367	0.485
**3D+FP**	HistGB	0.878	0.671	0.206	0.652	0.062	0.412	0.663	0.690	0.055	0.226	0.406
**3D+FP**	ExtraTrees	0.884	0.566	0.318	0.592	0.064	0.400	0.762	0.747	0.042	0.180	0.426
**3D+FP**	SVM	0.850	0.617	0.233	0.588	0.063	0.455	0.716	0.752	0.053	0.256	0.441
**3D+FP**	KNN	0.742	0.581	0.161	0.593	0.039	0.597	0.749	0.748	0.039	0.405	0.537
**3D+FP**	MLP	0.862	0.512	0.350	0.539	0.082	0.437	0.808	0.794	0.058	0.246	0.492
**3D+FP**	ElasticNet	0.783	0.667	0.116	0.653	0.038	0.547	0.667	0.690	0.027	0.398	0.493
**3D+FP**	PLS	0.547	0.530	0.016	0.522	0.031	0.792	0.793	0.812	0.032	0.591	0.602
**3D+FP**	Ridge	0.883	0.624	0.259	0.483	0.107	0.402	0.710	0.838	0.070	0.188	0.412
**3D+FP**	Stacking	0.852	0.682	0.170	0.693	0.039	0.452	0.652	0.646	0.057	0.300	0.448
**Combined**	RandomForest	0.852	0.677	0.175	0.663	0.038	0.452	0.657	0.680	0.032	0.286	0.431
**Combined**	XGBoost	0.821	0.678	0.143	0.661	0.039	0.497	0.656	0.682	0.031	0.358	0.464
**Combined**	LightGBM	0.849	0.678	0.171	0.667	0.044	0.457	0.657	0.676	0.037	0.297	0.434
**Combined**	GradientBoosting	0.825	0.669	0.156	0.666	0.044	0.492	0.666	0.677	0.036	0.352	0.475
**Combined**	HistGB	0.880	0.666	0.214	0.644	0.058	0.408	0.669	0.699	0.048	0.216	0.397
**Combined**	ExtraTrees	0.884	0.576	0.309	0.613	0.042	0.400	0.754	0.729	0.025	0.180	0.420
**Combined**	SVM	0.842	0.612	0.230	0.575	0.069	0.467	0.720	0.764	0.058	0.304	0.474
**Combined**	KNN	0.736	0.560	0.176	0.594	0.038	0.604	0.767	0.748	0.037	0.414	0.551

**Combined**	MLP	0.872	-37557744.304	37557745.176	0.476	0.088	0.420	7089.578	0.847	0.062	0.228	431.109
**Combined**	ElasticNet	0.828	-4605899.054	4605899.883	0.648	0.050	0.487	2482.721	0.695	0.037	0.338	151.275
**Combined**	PLS	0.542	-1188.763	1189.304	0.518	0.031	0.796	39.903	0.816	0.033	0.597	3.023
**Combined**	Ridge	0.883	-13060820.929	13060821.812	0.496	0.094	0.402	4180.766	0.829	0.060	0.189	254.373
**Combined**	Stacking	0.847	0.683	0.164	0.686	0.036	0.460	0.651	0.654	0.053	0.306	0.434
